# Development of a glutamine-responsive MRI contrast agent

**DOI:** 10.1039/d5sc05987a

**Published:** 2025-11-20

**Authors:** Charles A. Wilson, Austin T. Bruchs, Saman Fatima, David G. Boggs, Jennifer Bridwell-Rabb, Lisa Olshansky

**Affiliations:** a Department of Chemistry, University of Illinois Urbana-Champaign 600 S. Mathews Ave Urbana IL 61801 USA; b Department of Chemistry, University of Michigan, 930 N. University Ave Ann Arbor MI 48109 USA; c Center for Biophysics and Quantitative Biology, Chemical and Biomolecular Engineering, Materials Research Laboratory, and the Beckman Institute for Advanced Science and Technology, University of Illinois Urbana-Champaign 600 S. Mathews Ave Urbana IL 61801 USA lolshans@illinois.edu

## Abstract

Magnetic resonance imaging (MRI) is widely used to visualize disease, and image quality can be improved through use of MRI contrast agents. Currently available agents produce a signal based solely on spatial distribution, but modern metabolic profiling has uncovered a variety of biomarkers for disease. For example, tumors greatly increase their uptake and catabolism of glutamine (Gln), leading to modified local concentration. Our laboratory previously developed a switchable artificial metalloprotein (swArM) platform in which Gln-binding causes a protein conformational change that modifies the physicochemical environment of an installed metallocofactor. Installing MRI-active metallocofactors within swArMs, we present a proof-of-concept approch toward the development of an analyte-responsive MRI contrast agent. To develop these swArMs, we tested several MRI-active metals (Gd^3+^, Dy^3+^), chelating ligands (DOTA, DTPA, NOTA), and attachment sites, as well as the impacts of peripheral mutations on the Gln-responsive signal. In each case, metal content was analytically defined, and Gln-binding affinity was determined by isothermal titration calorimetry. Circular dichroism was used to verify that our swArMs could still undergo the conformational change. X-ray diffraction structures of the *apo-* and *holo-*swArMs further revealed that the metallocofactor is significantly solvent-exposed in both conformations, but exhibits additional interactions with the protein in the *holo-*state coinciding with the observed increase in *T*_2_ relaxivity of ∼60% upon Gln-binding.

## Abstract 2

This report details the development of a conformationally switchable artificial metalloprotein (swArM) that provides differential MRI contrast signal in the presence and absence of the key biomarker glutamine.

## Graphical abstract

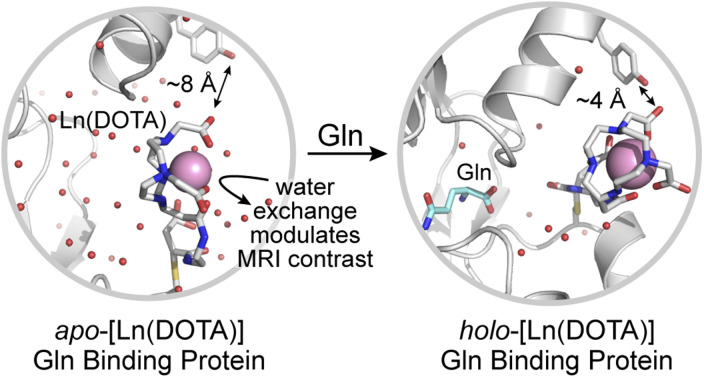

## Introduction

One of the hallmarks of cancer cell metabolism is the dysregulation of nutrient uptake, which is necessary to fuel the uncontrolled growth of tumors.^1^ A common form of this dysregulation is “glutamine addiction”, the phenomenon where cancerous cells greatly increase their uptake and catabolism of the amino acid glutamine (Gln).^2^ This change results in Gln concentration differences between diseased and healthy tissues.^3,4^ Direct sampling of tissue to measure these concentrations accurately is challenging and invasive,^5^ so methods to noninvasively assess Gln concentrations *in vivo* are desirable.

Most techniques used to characterize Gln addiction focus on studying Gln uptake and metabolic utilization rather than measuring concentration.^6–8^ The one exception is magnetic resonance spectroscopy (MRS), which is performed using the same instrument as the more commonly known magnetic resonance imaging (MRI). This technique allows spatial quantification of certain metabolites from their spectra.^9^ However, quantifying Gln is particularly challenging due to the extensive spectral overlap between Gln and glutamate (Glu). Even though this method does see some clinical use, it is recognized that the technique needs improvement, as it requires long imaging times and has issues with reproducibility, particularly at the field strengths typically used in the clinic (<3 T).^10^ An alternative to absolute quantification would be a relative imaging signal, highlighting areas where Gln concentration is in the diseased range. In this paper, we present a proof-of-concept for such a Gln-sensitive MRI contrast agent.

Standard MRI works by exciting the ^1^H nuclei of water molecules in tissues and measuring the resultant magnetic relaxation.^11^ This relaxation occurs in two modes: *T*_1_ relaxation (spin-lattice or longitudinal) and *T*_2_ relaxation (spin–spin or transverse). These relaxation rates are determined by the physicochemical environment surrounding the water ^1^H nuclei, so different tissues will appear brighter or darker on the image. These differences can be enhanced through the use of contrast agents, which shorten relaxation times and either brighten (*T*_1_-weighted) or darken (*T*_2_-weighted) regions in the image. All clinical contrast agents (and most experimental contrast agents in the literature) highlight diseased or damaged tissue simply through spatial distribution.^12^ The contrast agent either localizes to specific tissues or organs based on general chemical properties,^13^ or localizes to a molecular target due to an attached binding moiety.^14^ However, there are notable examples of “switchable” MRI contrast agents, where the MRI signal varies in response to a particular stimulus.^12^ Triggers include pH,^15,16^ redox potential,^17^ temperature,^18^ enzymatic activity,^19^ electrical activity during epileptic events,^20^ and binding of an ion^21–23^ or small molecule metabolite.^24–26^ The mechanisms leading to the change in MRI signal include conformational change,^27^ cleavage^28^ or protonation^29^ of side chains, aggregation^30^ or disaggregation^31^ of nanoparticles, change in the oxidation,^32^ or magnetic^22^ state of the MRI-active metal center.^33^

All FDA-approved MRI contrast agents are small molecules^34^ or nanoparticles,^35^ but the development of protein-based MRI contrast agents is an active area of study.^36–38^ Proteins can be designed to bind metals directly^39^ or, more commonly, a preexisting MRI contrast agent can be functionalized to bind to a protein^40,41^ to provide enhanced relaxivity and/or additional functionality. A contrast agent's effectiveness (termed “relaxivity”) depends on its interaction with water molecules, which are typically separated into inner sphere (binding directly to the metal center), second sphere (water molecules binding stably near the metal) and outer sphere (diffusing into and then out of the metal's magnetic field). Metalloproteins are known to stabilize second-sphere water molecules around the metallocofactor,^42^ potentially enhancing relaxivity in protein-based contrast agents.^37^ Another factor influencing the contrast agent's relaxivity is its rotation rate, which is dependent on the size of the molecule.^11^ Small molecules typically tumble too rapidly for optimal relaxation, so attaching a contrast agent to a slowly-tumbling macromolecule such as a protein can provide a significant boost to relaxivity.

**Fig. 1:**
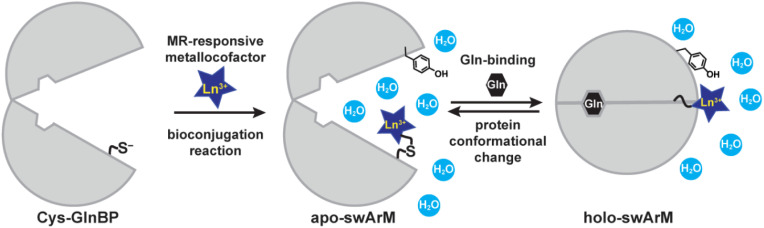
Conformationally switchable artificial metalloproteins (swArMs) are prepared through covalent attachment of a metallocofactor to a single-Cys variant of the glutamine-binding protein (GlnBP) from *E. coli*. Upon binding Gln, the protein undergoes a conformational change that modifies the interactions between the metallocofactor, protein, and surrounding water molecules. Changes in metallocofactor mobility and water association alter the contrast signal during MRI measurements, providing a method with which to assess Gln concentrations *in vivo*.

We previously reported switchable artificial metalloproteins (swArMs) that were constructed by attaching a metal complex (stoichiometrically and site-specifically) to the *E. coli* Gln-binding protein (GlnBP).^43^ The resultant constructs exhibited distinct changes in hydrogen-bonding (H-bonding) interactions around the installed metallocofactors upon going from *apo-* to *holo-*conformations when Gln binds.^43,44^ Taking advantage of these advances, we report installation of the known lanthanide (Ln^3+^) chelating ligand, dodecane tetraacetic acid (DOTA), into GlnBP (Fig. 1). We examine the relaxivity of these new swArM constructs as a function of bioconjugation site, nature of the DOTA-derived ligand, Ln^3+^ speciation, and modification of the surrounding microenvironment though mutagenesis. From these studies, we provide an innovative and modular approach to generating switchable analyte-responsive protein-based MRI contrast agents.

## Results

### Preparation of MRI-active swArMs

**Chart 1:**
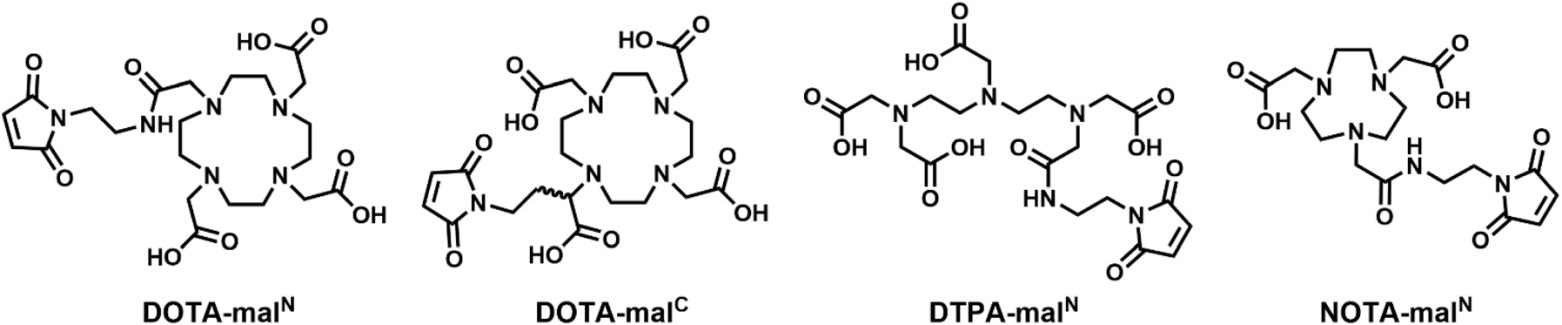
Chelating ligands used for bioconjugation and metalation to create swArMs.

Building from methods previously established in our laboratory,^43^ swArMs were prepared from single-Cys variants of GlnBP by site-specific covalent attachment of maleimide-functionalized chelating ligands (Chart 1), metalated with MRI-active Ln^3+^ ions as detailed in the SI. The extent of bioconjugation and metalation were first assessed using liquid chromatography-mass spectrometry (LC-MS). Here, our optimized protocol described in the SI provided near-complete bioconjugation (Fig. S1–S5), with the exception of small amounts (<10%) of unconjugated protein containing irreversibly oxidized cysteine sulfinate residues, evidenced by the +31 Da mass shift for Cys-GlnBP variants (Fig. S2, leftmost peaks). The LC-MS results also showed that all proteins functionalized with DOTA were quantitatively metalated. Since MRI contrast signal is directly related to metal ion concentration, we further quantified the metal content in our swArMs by energy-dispersive X-ray fluorescence (EDXRF) spectroscopy. This element-specific method, along with protein concentrations determined by UV-vis absorption spectroscopy, revealed that the swArMs were generally 90–95% metalated (Fig. S6). Notably, all relaxivity data are reported relative to measured Ln^3+^ concentrations by EDXRF, which were determined uniquely for each sample preparation.

The introduction of metallocofactors within GlnBP bears the risk of compromising the protein's “switchability”. To determine the effect of bioconjugation on protein function, isothermal titration calorimetry (ITC) was used to assess Gln binding, and circular dichroism (CD) spectroscopy was used to observe the resultant conformational change. ITC data were fit using an independent binding model plus a linear function to account for background heat released.^45^ In this work, we examined several variants of GlnBP swArMs. ITC data are shown in Fig. S7–S21, and *K*_d_s for Gln are listed in Table S1. Bioconjugation caused a ∼three-fold increase in Gln dissociation constants for the main variant studied (T_72_C-GlnBP), with the *K*_d_ increasing from 0.6 ± 0.1 µM to 1.8 ± 0.8 µM on bioconjugation with Gd(DOTA-mal^N^). However, with expected physiological Gln concentrations of 0.4–0.8 mM,^2^ the tight binding of our constructs (regardless of the increase in *K*_d_) would result in the protein always being in the *holo-*state. Notably, there are published mutations in the GlnBP ‘hinge region’ which could be employed to modulate the dynamic range of Gln-binding for future *in vivo* applications.^46^ Other variants had a one- to four-fold increase in *K*_d_, with the exception of the N_160_C-GlnBP variant, which had a ∼1000-fold increase (Fig. S12 and S13).

We investigated the T_72_C and N_160_C variants further *via* CD spectroscopy. The CD spectrum across 260–320 nm reflects tertiary structure, so by monitoring spectral changes upon addition of a saturating concentration of Gln we could detect protein conformational changes. Fig. S22 shows that the CD spectral changes for *apo-* and *holo-*T_72_C-GlnBP are similar to those of *apo-* and *holo-*[Gd(DOTA-mal^N^)]_72_-GlnBP (where the subscript following square brackets indicates the site of metallocofactor attachment). In contrast, the CD spectra of T_160_C-GlnBP and [Gd(DOTA-mal^N^)]_160_-GlnBP (Fig. S23) reveal that conjugation of Gd(DOTA-mal^N^) within this variant severely hinders tertiary structural change.

### Relaxivity measurements

**Fig. 2:**
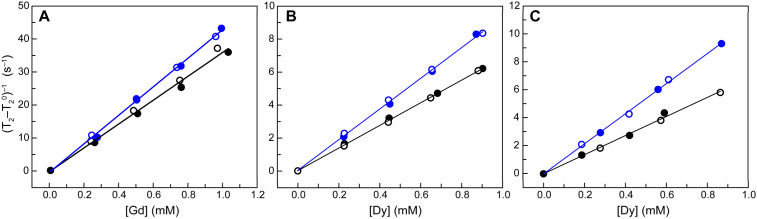
*T*_2_ relaxivity data at 9.4 T for *apo-* (black) and *holo-* (blue) [Gd(DOTA-mal^N^)]_72_-GlnBP (A), [Dy(DOTA-mal^N^)]_72_-GlnBP (B), and [Dy(DOTA-mal^N^)]_72_-A_126_Y-GlnBP (C). Open and filled circles represent replicate sets of experiments performed on independently prepared samples in PBS buffer, pH 7.4, 37 °C.

The effectiveness of a contrast agent depends on its “relaxivity”, which is measured in units of mM^−1^ s^−1^, and quantifies how much a given concentration of the contrast agent improves water relaxation rates. *T*_1_ and *T*_2_ relaxivities are denoted *r*_1_ and *r*_2_, respectively, and are calculated according to eqn (1):1
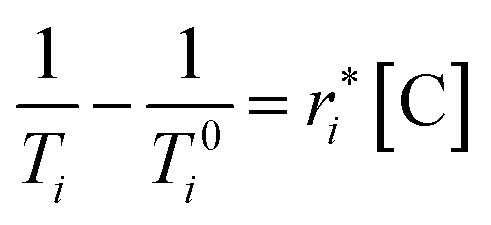
Here, *T*_*i*_ and *T*_*i*_^0^ are the relaxation times in the presence and absence of the contrast agent, respectively, and [C] is the concentration of the contrast agent. As shown in Fig. 2, the value of *r*_*i*_ is taken from the slope of the line obtained by measuring *T*_*i*_ values as a function of concentration. The relaxivity of a contrast agent depends not only on the properties of the molecule but also on the strength of the magnetic field used for the measurement. Whereas early MRI instruments were relatively weak (<1 T), the modern clinical standard has risen to 1–3 T, wth an increasing number of even more powerful instruments (>3 T) in recent years. Thus, we performed relaxivity experiments at both the most clinically relevant field strengths 1.4 T and at the more cutting-edge field strength of 9.4 T.

[Gd(DOTA-mal^N^)]_72_-GlnBP (see Chart 1 for ligand names) was chosen as the first variant to study, as [Gd(DOTA)(H_2_O)]^−^ is one of the most commonly used contrast agents,^12^ and in previous work from our laboratory, swArMs prepared in the T_72_C-GlnBP variant exhibited the greatest changes in H-bond interactions between the *apo-* and *holo-*states.^43^ As shown in Table 1, S2, and S3, *apo-*[Gd(DOTA-mal^N^)]_72_-GlnBP has a high *r*_1_ at 1.4 T (19.5 ± 0.4 mM^−1^ s^−1^) and a moderate *r*_1_ at 9.4 T (4.35 ± 0.07 mM^−1^ s^−1^). *r*_2_ is high at both field strengths (37.2 ± 0.7 and 36 ± 1 mM^−1^ s^−1^, respectively). Compared to Gd(DOTA), these values represent a ∼six-fold increase in *r*_1_ at 1.4 T and a ∼10-fold increase in *r*_2_ at both field strengths.^47^ This enhanced relaxivity can be explained by the slower rotation rate for a protein compared to a free ligand, but might also include contributions from increased second sphere water interactions. Upon Gln- binding, there is negligible change in *r*_1_ (1.4 T: 20.4 ± 0.3 mM^−1^ s^−1^, 9.4 T: 4.32 ± 0.07 mM^−1^ s^−1^), but an increase in *r*_2_ of 11 ± 2% at 1.4 T (41.5 ± 0.4 mM^−1^ s^−1^) and 20 ± 5% at 9.4 T (43.2 ± 0.8 mM^−1^ s^−1^) as shown in Table 1 and Fig. 2, S24–S26.

Relaxivity values (mM^−1^ s^−1^) of *Apo-* and *Holo-*swArMs^a^

**Table:** 

swArM^b^ (Field Strength)	*r*_1_ (*Apo*)	*r*_1_ (*Holo*)	*Holo*/*Apo* % *r*_1_ Change	*r*_2_ (*Apo*)	*r*_2_ (*Holo*)	*Holo*/*Apo* % *r*_2_ Change
[Gd(DOTA-mal^N^)]_72_ (1.4 T)	19.5 ± 0.4	20.4 ± 0.3	4 ± 2	37.2 ± 0.7	41.5 ± 0.4	11 ± 2
[Gd(DOTA-mal^N^)]_72_ (9.4 T)	4.35 ± 0.07	4.32 ± 0.07	–1 ± 2	36 ± 1	43.2 ± 0.8	20 ± 5
[Dy(DOTA-mal^N^)]_72_ (9.4 T)	0.37 ± 0.01	0.37 ± 0.02	–1 ± 5	6.83 ± 0.04	9.22 ± 0.09	36 ± 3
[Dy(DOTA-mal^N^)]_72_-N_127_Y (9.4 T)	—	—	—	6.5 ± 0.3	9.1 ± 0.2	40 ± 7
[Dy(DOTA-mal^N^)]_72_-A_126_Y (9.4 T)	—	—	—	6.8 ± 0.2	10.8 ± 0.2	58 ± 6

a Uncertainty limits of *r*_i_ represent the standard error of the fit. Uncertainty limits of the percentage change in *holo*/*apo* relaxivity are from propagation of error in each individual value (*apo* and *holo*).

b Determined from two sets of independently prepared samples.

c Determined from one set of independently prepared samples. All samples were prepared in PBS buffer, at pH 7.4, and data were collected at 37 °C for 9.4 T, and 32 °C for 1.4 T measurements.

**Table:** 

Contrast Agent^c^ (Field Strength)	*r*_1_ (−Gln)	*r*_1_ (+Gln)	+Gln/−Gln % *r*_1_ Change	*r*_2_ (−Gln)	*r*_2_ (+Gln)	+Gln/−Gln % *r*_2_ Change
Gd(DOTA) (9.4 T)	2.82 ± 0.04	2.80 ± 0.07	0 ± 3	3.5 ± 0.1	3.6 ± 0.2	1 ± 6
Dy(DOTA) (9.4 T)	0.21 ± 0.02	0.211 ± 0.004	0 ± 9	0.99 ± 0.02	1.02 ± 0.02	3 ± 3

### Enhancing *holo*/*apo* relaxivity differences

With these initial measurements in hand, we set out to develop variants with enhanced *holo*/*apo* relaxivity differences. To these ends, we modified the site of attachment of the metallocofactor reagent, the ligand used to prepare the metallocofactor, the metal used to prepare the metallocofactor, and the influence of peripheral mutations.

### Optimizing the site of attachment

To investigate alternative attachment sites, we selected two additional variants. In the *holo-*state, N_160_C is located deeper in the protein interior relative to T_72_C, while D_122_C is located further toward the surface.^43,44^ In addition to expected changes in solvent accessibility, each attachment site on the protein has the potential for unique interactions with local sidechains. Intriguingly, changing the attachment site had a dramatic effect on *r*_2_ (Table S3, Fig. S27 and S28). Compared to [Gd(DOTA)mal^N^)]_72_-GlnBP, *r*_2_ was doubled for the N_160_C variant (80 mM^−1^ s^−1^) and halved for the D_122_C variant (18 mM^−1^ s^−1^). This change illustrates the importance of the local protein environment on determining relaxivity. However, in both cases the *holo*/*apo* change was small (<10%), so they proved to be unsuitable for our application. For the N_160_C variant, this small change is presumably due to the lack of conformational change, as revealed by CD spectroscopy (discussed above). For the D_122_C variant, it likely indicates that the conformational change of the protein does not alter the protein-metallocofactor interactions. From these results, we concluded that T_72_C-GlnBP outperformed the others and selected it as our platform moving forward.

### Varying the chelating ligand

Next, we set out to investigate alternative ligands to coordinate Ln^3+^ ions. As shown in Chart 1, we investigated two DOTA derivatives (DOTA-mal^N^ and DOTA-mal^C^) as well as an acyclic version of DOTA, diethylene triamine pentaacetic acid (DTPA), and a six-coordinate version of the eight-coordinate DOTA ligand, nonane triacetic acid (NOTA). When solvated outside of a protein complex, DTPA exhibits slightly higher relaxivities at the cost of kinetic stability.^48^ In our hands, *apo-*[Gd(DTPA-mal^N^)]_72_-GlnBP had an increased *r*_2_ (65 mM^−1^ s^−1^, 9.4 T) relative to *apo-*[Gd(DOTA-mal^N^)]_72_-GlnBP (Table S3, Fig. S29), but only exhibited a ∼10% change in *r*_2_ on Gln binding. We notably did not observe loss of metal to solution over time in this system. In contrast, samples of [Gd(NOTA)mal^N^)]_72_-GlnBP gave rise to precipitate formation upon preparation for relaxivity studies in PBS buffer. This ligand has previously been tested with Gd^3+^ as an MRI contrast agent in PBS buffer, with no reported stability issues.^40^ The fact that conjugation to our protein decreased the kinetic stability of Gd(NOTA) exemplifies the impact of the specific protein environment. Unfortunately, in this case the specific protein environment proved detrimental to our goals.

Finally, we tested an alternative functionalization of the DOTA ligand. The more commonly used version attaches the maleimide linker by converting a carboxyl group into an amide group (mal^N^, Chart 1). A different option is to functionalize the carboxylate arm at carbon (mal^C^, Chart 1), leaving all four carboxylate arms intact in a similar manner to unmodified DOTA. Reasoning that the difference in coordination, and/or the linker length (the length between the maleimide functional group and the chelating macrocycle is three atoms for mal^C^*vs.* five atoms for mal^N^) could lead to differences in relaxivity,^49^ we also tested this chelating agent. Unfortunately, the *holo*/*apo* enhancement was insignificant (Table S3 and Fig. S30). Thus we continued with DOTA-mal^N^ as our ligand of choice.

### Exploring the impacts of Ln^3+^ identity

We next explored an alternative MRI-active metal, Dy^3+^. Since the advent of MRI, there has been a constant drive for higher field strengths, as this improves the signal-to-noise ratios and hence the quality of images.^50^ The *T*_2_ relaxivities of Dy^3+^-based contrast agents primarily arise from the Curie mechanism (discussed further below), which increases as the square of magnetic field strength.^51^ Accordingly, we investigated the impacts of the metal speciation on relaxivity in our swArMs at high field strengths. We found that, as expected, the *r*_1_ value of [Dy(DOTA-mal^N^)]_72_-GlnBP was below the practical detection limit (Table 1 and Fig. S31). However, *r*_2_ was 6.86 ± 0.08 mM^−1^ s^−1^ (*apo*) and 9.3 ± 0.1 (*holo*). Although these relaxivites are lower than those of the equivalent Gd-swArM, the *holo*/*apo* enhancement is higher than that of [Gd(DOTA-mal^N^)]_72_-GlnBP (36 ± 3%, *versus* 20 ± 5%, Table 1 and Fig. 2) and thus is preferable for our purposes. As Dy^3+^ only provides appreciable relaxivity at 9.4 T, we ceased performing experiments at 1.4 T and moved forward to investigate the impacts of peripheral mutations on the relaxivity of [Dy(DOTA-mal^N^)]_72_-GlnBP.

As controls, we also measured the relaxivities of unmodified Gd(DOTA) and Dy(DOTA) in the presence and absence of Gln. The presence of Gln did not appreciably change relaxivity values (Table 1 and Fig. S32), and the measured values are consistent with those reported in the literature.^47,52^

### Installing mutations around the metallocofactor

In previous work, we applied a combination of single crystal X-ray diffraction (XRD), molecular dynamics simulations, and FTIR spectroscopy to develop variants of T_72_C-functionalized swArMs exhibiting enhanced changes in H-bonding interactions in their *apo-* and *holo-*states.^44^ From these studies G_119_K, A_126_Y, N_127_Y, and Q_183_K were all shown to modulate the H-bonding interactions around the installed metallocofactor upon Gln-binding. This series of T_72_C double variants were purified, bioconjugated with Dy(DOTA-mal^N^), and their relaxivities were assessed. Gratifyingly, these mutations successfully enhanced the *holo*/*apo* relaxivity ratios in some cases (Table 1, S4 and Fig. 2, S33–S37). The A_126_Y variant had the same *apo-r*_2_ as the single variant (6.8 ± 0.2 mM^−1^ s^−1^), while the *holo-r*_2_ was increased (10.8 ± 0.2 mM^−1^ s^−1^). This difference resulted in the largest Gln-responsive enhancement of all studied variants, providing a 58 ± 6% enhancement in the *holo*/*apo r*_2_ ratio (Table 1).

### Single crystal X-ray diffraction

Using our previously established conditions, we were able to prepare diffraction quality crystals of *apo-*[Gd(DOTA-mal^N^)]_72_-GlnBP.^44^ However, the *holo-*[Gd(DOTA-mal^N^)]_72_-GlnBP crystallization conditions required optimization.^43^ Accordingly, we screened 384 crystallization conditions. Optimization around the best candidate produced the same 0.8 mm × 0.1 mm × 0.1 mm needle-like crystals previously prepared for *holo-*swArMs.^43^ Separate XRD datasets for *apo* (PDB 9P4E) and *holo* (PDB 9P4D) crystals were collected at beamline 12–2 located at the Stanford Synchrotron Radiation Lightsource (SSRL).^53–56^ Phases were obtained using molecular replacement and the previously published *holo*-swArM structure (PDB : 8EYZ) as a search model.^43^ More detailed methods for data collection, processing, phasing, and structural refinement can be found in the SI.

**Fig. 3:**
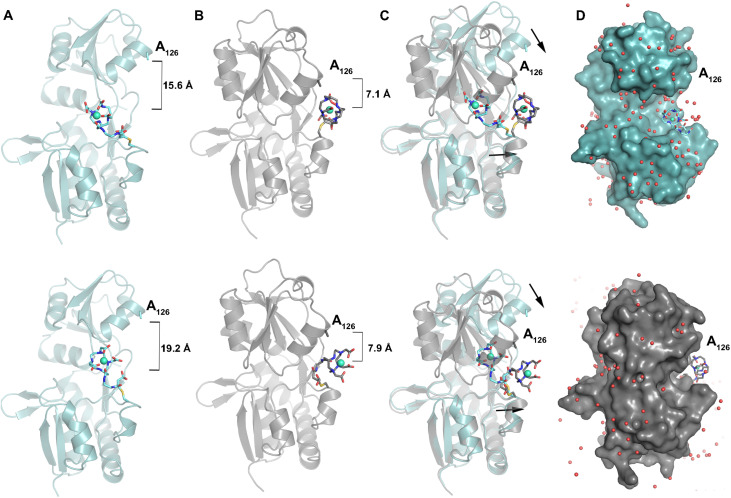
Conformational change upon Gln binding displaces the [Gd(DOTA-mal^N^)] metallocofoactor. (A) Without Gln (PDB 9P4E), the protein scaffold adopts the *apo-*conformation, and [Gd(DOTA-mal^N^)] has limited interactions with the protein. [Gd(DOTA-mal^N^)] is far away from A_126_. (B) With Gln bound (PDB 9P4D), the protein adopts a *holo-*conformation, displacing the [Gd(DOTA-mal^N^)] metallocofactor closer to A_126_. (C) An overlay of each conformation highlights the closing of the protein scaffold and displacement of [Gd(DOTA-mal^N^)]. The top and bottom images of panels A–C illustrate two modeled orientations of [Gd(DOTA-mal^N^)] from one exemplary chain (chain C). (D) The modeled conformations of the metallocofactor are extremely solvent exposed in the *apo-*protein (top panel). In the *holo-*protein structure, the Gln induced conformational change results in changes to the microenvironment of [Gd(DOTA-mal^N^)] (bottom panel, Chain C). In panels A–B, measurements reflect the distance between the sidechain of A_126_ and Gd^3+^. Additional information regarding anomalous maps used to model the alternate conformations and H-bonding networks with Gln can be found in Fig. S40–S43.

The overall protein folds of *apo-* and *holo-*[Gd(DOTA-mal^N^)]_72_-GlnBP are not significantly different from those of WT-GlnBP (r.m.s.d. of 0.824 and 0.579 Å for 1404 and 1402 atoms, respectively).^57,58^ We observe one protein molecule per asymmetric unit for *apo-*[Gd(DOTA-mal^N^)]_72_-GlnBP, and six for *holo-*[Gd(DOTA-mal^N^)]_72_-GlnBP. For both the *apo-* and *holo-*swArM structures, we note that four possible isomers may form upon bioconjugation with Gd(DOTA-mal^N^). The structures of these isomers are shown in Fig. S38. Here nucleophilic attack of Cys_72_ on the maleimide olefin can give rise to two regioisomers, each with two different enantiomeric orientations. Reflective of this complexity, and the electron density maps, two metal complexes representing the two regioisomers were modeled (Fig. S39, S43 and S44). In both complexes, the Cys-S–C-maleimide bond was modeled as planar to represent both enantiomers of each regioisomer. Thus, although chemically incorrect, this structure solution provides the best balance between over- and under-fitting the data. It is important to be aware that as a result of this structural inhomogeneity and the observed flexibility of the incorporated ligands, the data lack sufficient detail to comment on the precise coordination environments of Gd^3+^ in each swArM. Notwithstanding, the general globular placement of the metallocofactors in each structure are extremely informative (Fig. 3).

In the *apo-*structure, the modeled conformations of the metallocofactor are highly solvent exposed, with many crystallographic waters in the space between it and the protein (Fig. 3A and D). The linker is lying against the surface of the protein, and the solid-state interactions between the chelator and the protein appear to be limited to van der Waals contacts between carbons in the macrocycle and the backbone oxygen of G_117_.

The position and orientation of Gd(DOTA-mal^N^) cofactors in the *holo-*protein structure, which contains one molecule of Gln per chain, show significant variation, but there are consistent patterns relevant to modulation of MRI contrast agent relaxivity (Fig. S40 and S41). We can divide the twelve modeled metallocofactors into two groups by proximity to A_126_ (measured between Gd^3+^ and the side chain carbon of A_126_). Although A_126_ does not interact with Gd(DOTA-mal^N^), it is a convenient reference point, and mutation at this site enhances relaxivity. Five of the modeled metallocofactors are closer to A_126_ (6.8–7.9 Å), and seven are more distant (10.3–14.1 Å) (Fig. S40). Of the five adjacent to A_126_, three are turned directly toward the protein (Fig. 3B, S40A and C). Conversely, the seven metallocofactors more distant from A_126_ are oriented away from the protein and assume a conformation that resembles the ligand in the bottom panel of Fig. 3B, and shown in Fig. S40B and D–F. In this conformation, the solid state interactions between the ligand carboxylates and the protein are comparatively reduced or absent. In all 12 cases, there is notably closer proximity between the protein and the linker, and in many cases between the protein and the macrocycle of the DOTA-mal^N^ chelate (Fig. 3D).

### Temperature-dependent relaxivity measurements

To better understand the mechanisms underlying relaxivity in swArMs, we investigated the temperature dependencies of their relaxivity values. As mentioned, the relaxation mechanisms at play in a given system depend heavily on the movement of the contrast agent in solution and its stable interactions with water molecules. Consequently, relaxivity varies as a function of temperature, with the exact relation dependent on the details of those interactions. In an effort to explain the observed *holo*/*apo r*_2_ enhancements of [Dy(DOTA-mal^N^)]_72_-GlnBP, we measured the temperature dependence of its relaxivity in the presence and absence of Gln (Fig. S45). For the *apo*-form, *r*_2_ values increase with decreasing temperature over the entire range studied. The *holo*-form follows the same pattern until approximately 15 °C, where it reaches a maximal value of ∼22 mM^−1^ s^−1^ before beginning to decrease. These data reveal that there is a clear shift in the curve towards higher temperatures on going from *apo*- to *holo*-swArM states. Potential implications for these findings are discussed below.

## Discussion

As our understanding of the biochemical markers for disease has improved, there has been a drive to improve diagnostics and treatment by directly imaging these indicators.^59^ However, all MRI contrast agents that are currently commercially available provide contrast through the distribution of a static agent throughout the body.^12^ One method for localized detection of biomarkers is to create a “dynamic” MRI contrast agent, which modifies its signal in the presence of said biomarker.

Previously, our laboratory developed swArMs which, upon binding Gln, modified the H-bonding network surrounding an installed cobaloxime metallocofactor.^43,44^ Owing to the sensitivity of MRI contrast agents to their surrounding H-bond interactions, we have now taken advantage of these Gln-dependent conformational changes by installing MRI contrast agents within swArMs. In this way, we have created the first iteration of a Gln-responsive MRI contrast agent. While no variant showed a significant *holo*/*apo* difference in *r*_1_, Gln binding caused a 20 ± 5% enhancement in *r*_2_ at 9.4 T for our first construct. We were able to improve this enhancement to 36 ± 3% by changing the metal to Dy^3+^, and increased it further to 58 ± 6% by introducing the peripheral mutation A_126_Y.

Detailed measurement and analysis of the factors determining the relaxivities are beyond the scope of this work, but there are a few well-established principles that can shed light on our results. It is often assumed that the identity of the bound Ln^3+^ does not affect the occupancy of waters interacting with the contrast agent.^60^ If we adopt the same assumption then the difference in relaxivity upon Gln binding in the Gd^3+^- and Dy^3+^-swArMs requires another explanation. There are three different relaxation mechanisms which come into play at the magnetic field strengths used in this work: dipolar relaxation, scalar relaxation, and Curie relaxation.^12^ As a consequence of its slow electronic relaxation, Gd^3+^ does not act appreciably *via* the Curie mechanism. Instead, dipolar effects commonly contribute most strongly to the relaxation mechanism for this metal ion, with scalar relaxation typically smaller, though not always non-negligible. Meanwhile, Dy^3+^ acts primarily *via* the Curie mechanism, with scalar relaxation being negligible and dipolar relaxation contributions being small.^51^ Each mechanism has a distinct dependence on the properties of the contrast agent (for a detailed mathematical description see eqn. 3–8 in ref. 14). Thus it is unsurprising that the same population of water molecules can give a 19% *holo*/*apo* enhancement in the Gd^3+^-swArM and a 35% enhancement in the Dy^3+^-swArM.

To the best of our knowledge, there are no published crystal structures of [Gd(DOTA-mal^N^)], but there are structures of both unmodified Na[Gd(DOTA)(H_2_O)],^61^ and [Gd(DOTA)]^−^ functionalized with a different linker bound to a protein.^62^ In both structures Gd^3+^ binds a single inner sphere water molecule. While the existence of this inner sphere water is verified by both NMR and fluorescence spectroscopies in other [Ln(DOTA)(H_2_O)]^−^ complexes,^60,63^ we are unaware of any similar published data for [Ln(DOTA-mal^N^)]^−^ complexes. However, as there is no reason to suspect that the complex lacks an inner sphere water, and literature reports assume its presence without comment,^41,64^ we assume that the absence of an inner sphere water in our XRD structures is merely an artifact of the ambiguity resulting from heterogeneous constructs present in the crystal structures.

In the crystal structures, we observe that in the *apo-*state the linker has relatively few interactions with the protein, and the metallocofactor is completely solvent exposed (Fig. 3A and D). In the *holo-*state, the linker has increased contact with the protein (Fig. 3B and D). Although Gd(DOTA-mal^N^) does not interact with residues A_126_ and N_127_, the crystal structure explains the pattern of relaxivity enhancement caused by the peripheral mutations: A_126_D < N_127_Y < A_126_Y. The side chains are oriented toward Gd(DOTA-mal^N^), with the former being ∼5 Å from the closest atom and the latter being slightly further away despite having a longer side chain. Thus, the side chain of A_126_Y may be closer to the metallocofactor than N_127_Y, and be able to interact more strongly. Meanwhile, the significantly shorter side chain of A_126_D may not reach the metallocofactor to interact effectively.

Our temperature-dependent relaxivity experiments reveal that at 37 °C, swArMs are well below their maximum relaxivity. *Holo-*[Dy(DOTA-mal^N^)]_72_-GlnBP shows an inflection point at 15 °C, with a maximal relaxivity of ∼22 mM^−1^ s^−1^. The shape of the curves for these *apo* and *holo*-swArMs suggest that binding Gln causes the relaxivity curve to shift towards a higher temperature. Introducing additional mutations could potentially shift the peak further to enable clinically relevant *in vivo* Gln imaging extending beyond differences in contrast agent biodistribution gradients.

While much work remains to transform these proof-of-concept systems into clinically relevant contrast agents, the present report signifies a significant departure from other published systems. Here, the specificity of GlnBP allows it to report on a single biomarker to the exclusion of chemically similar metabolites such as Glu. This approach differs from excellent advances made by Jasanoff *et al*. in which analyte-responsive protein contrast agents were developed through directed evolution.^25,26^ These systems require a new directed evolution campaign for each analyte to be studied.^65^ In contrast, there are numerous structurally related periplasmic binding proteins that each bind to unique biologically relevant small molecules with high specificity. These analytes include arginine,^66^ ornithine,^67^ putrescine,^68^ and spermidine,^69^ to name a few, which have significantly different concentrations in healthy *vs.* diseased tissues.^70^ For *in vivo* applications, mutations in the “hinge” region of GlnBP (and other periplasmic binding proteins) can be installed to tune the dynamic range of binding to match physiological concentrations (0.4–0.8 mM for Gln),^2,46^ and strategies to avoid host immunogenic response will need to be implemented.^71^ However, the studies presented here illustrate our approach to the modular development of analyte-responsive MRI contrast agents.

## Conclusions

We have installed known MRI contrast agents within the periplasmic Gln-binding protein from *E. coli* to create novel analyte-responsive swArMs. Upon binding Gln, swArMs undergo a large-scale conformational change, modulating the H-bond interactions about the bound metallocofactor. Optimizing the site of attachment of the MRI contrast agent, its chelating ligand, the identity of the metal ion bound, and the impacts of peripheral mutations, we have optimized these swArMs to enhance the *holo*/*apo r*_2_ relaxivity ratios. We have structurally characterized these constructs by single crystal XRD in both conformational states and performed temperature-dependent relaxivity studies to illuminate potential opportunities for improvement. These studies provide a proof-of-concept for a new approach toward analyte-responsive MRI contrast agents that may one day be used in the early detection of diseased states.

## Author contributions

A. B. and D. B. collected and modelled the X-ray data. C. W., S. F., and N. M. D. performed all other experiments and data analysis. L. O. and J. B.-R. oversaw the project. L. O. conceived of the project. C. W. and L. O. wrote the manuscript. All authors participated in the discussion and preparation of the manuscript.

## Conflicts of interest

There are no conflicts to declare.

## Supplementary Material

SC-OLF-D5SC05987A-s001

## Data Availability

The data supporting this article have been included as part of the supplementary information (SI). Supplementary information: these data include materials and methods, *K*_d_s for Gln dissociation from variants, *r*_1_ and *r*_2_ values of variants, raw and fit relaxivity data, LC-MS spectra of variants, exemplary EDXRF quantification data, exemplary ITC data, CD spectra, *T*_1_ and *T*_2_ data, temperature-dependent relaxivity data, electron density maps for Gd^3+^, and H-bonding networks observed by XRD. See DOI: https://doi.org/10.1039/d5sc05987a.
