# Supplementary material for: Development of a glutamine-responsive MRI contrast agent
Source: Chem Sci. 2025 Nov 20;17(5):2781–90. doi: 10.1039/d5sc05987a (PMC12699829; doi:10.1039/d5sc05987a)
Supplement: SC-017-D5SC05987A-s001 [file SC-017-D5SC05987A-s001.pdf]

Supplementary Information (SI) for Chemical Science.
This journal is © The Royal Society of Chemistry 2025

Development of a Glutamine-Responsive MRI Contrast Agent

Charles A. Wilson,[a] Austin T. Bruchs,[b] Saman Fatima,[a] David G. Boggs,[b] Jennifer Bridwell-Rabb,[b] and Lisa Olshansky[a,c]*

[a]Department of Chemistry, University of Illinois Urbana-Champaign, 600 S. Mathews Ave. Urbana, IL 61801, United States.

[b]Department of Chemistry, University of Michigan, 930 N. University Ave. Ann Arbor, MI 48109, United States.

[c]Center for Biophysics and Quantitative Biology, Department of Chemical and Biomolecular Engineering, Materials Research Laboratory, and Beckman Institute for Advanced Science and Engineering, University of Illinois Urbana-Champaign, 600 S. Mathews Ave. Urbana, IL 61801, United States.

*lolshans@illinois.edu

Index .... *Page*

Materials S3
Physical Methods and Instrumentation S4
Table S1. Gln Dissociation Constants Assessed by ITC for swArM Variants S8
Table S2. Single-Mutant Relaxivities at 1.4 T S9
Table S3. Single-Mutant Relaxivities at 9.4 T S10
Table S4. Double-Mutant Relaxivities at 9.4 T S11
Table S5. XRD Processing and Refinement Statistics for [Gd(DOTA-mal^{N})]-GlnBP S12
Figure S1. LC-MS Trace of $T_{72}C$-GlnBP S13
Figure S2. LC-MS Trace of [Gd(DOTA-mal^{N})]$_{72}$-GlnBP S14
Figure S3. LC-MS Trace of [Dy(DOTA-mal^{N})]$_{72}$-GlnBP S15
Figure S4. LC-MS Trace of $T_{72}C$/$A_{126}Y$-GlnBP S16
Figure S5. LC-MS Trace of [Dy(DOTA-mal^{N})]$_{72}$-$A_{126}Y$-GlnBP S17
Figure S6. EDXRF Calibration Curve and Experimental Data for Gd Quantification S18
Figure S7. ITC Thermogram and Analysis for $T_{72}C$-GlnBP Binding Gln S19
Figure S8. ITC Thermogram and Analysis for [Gd(DOTA-mal^{N})]$_{72}$-GlnBP Binding Gln S20
Figure S9. ITC Thermogram and Analysis for [Gd(DTPA-mal^{N})]$_{72}$-GlnBP Binding Gln S21
Figure S10. ITC Thermogram and Analysis for [Gd(DOTA-mal^{N})]$_{122}$-GlnBP Binding Gln S22
Figure S11. ITC Thermogram and Analysis for $N_{160}C$-GlnBP Binding Gln S23
Figure S12. ITC Thermogram and Analysis for [Gd(DOTA-mal^{N})]$_{160}$-GlnBP Binding Gln S24
Figure S13. ITC Thermogram and Analysis for $T_{72}C$-$G_{119}R$-GlnBP Binding Gln S25
Figure S14. ITC Thermogram and Analysis for [Gd(DOTA-mal^{N})]$_{72}$-$G_{119}R$-GlnBP Binding Gln S26
Figure S15. ITC Thermogram and Analysis for $T_{72}C$-$A_{126}D$-GlnBP Binding Gln S27
Figure S16. ITC Thermogram and Analysis for [Gd(DOTA-mal^{N})]$_{72}$-$A_{126}D$-GlnBP Binding Gln S28
Figure S17. ITC Thermogram and Analysis for $T_{72}C$-$A_{126}Y$-GlnBP Binding Gln S29
Figure S18. ITC Thermogram and Analysis for [Gd(DOTA-mal^{N})]$_{72}$-$A_{126}Y$-GlnBP Binding Gln S30
Figure S19. ITC Thermogram and Analysis for [Gd(DOTA-mal^{N})]$_{72}$-$N_{127}Y$-GlnBP Binding Gln S31
Figure S20. ITC Thermogram and Analysis for $T_{72}C$-$Q_{183}Y$-GlnBP Binding Gln S32
Figure S21. ITC Thermogram and Analysis for [Gd(DOTA-mal^{N})]$_{72}$-$Q_{183}Y$-GlnBP Binding Gln S33
Figure S22. CD Spectra of *apo*/*holo*-$T_{72}C$-GlnBP and [Gd(DOTA-mal^{N})]$_{72}$-GlnBP S34
Figure S23. CD Spectra of *apo*/*holo*-$N_{160}C$-GlnBP and [Gd(DOTA-mal^{N})]$_{160}$-GlnBP S35

Figure S24. T_1 Relaxation Data and Analysis for *apo*/*holo*-[Gd(DOTA-mal^N)]$_{72}$-GlnBP at 1.4 T S36
Figure S25. T_1 Relaxation Data and Analysis for *apo*/*holo*-[Gd(DOTA-mal^N)]$_{72}$-GlnBP at 9.4 T S37
Figure S26. T_2 Relaxation Data and Analysis for *apo*/*holo*-[Gd(DOTA-mal^N)]$_{72}$-GlnBP at 1.4 T S38
Figure S27. T_2 Relaxation Data and Analysis for *apo*/*holo*-[Gd(DOTA-mal^N)]$_{122}$-GlnBP at 9.4 T S39
Figure S28. T_2 Relaxation Data and Analysis for *apo*/*holo*-[Gd(DOTA-mal^N)]$_{160}$-GlnBP at 9.4 T S40
Figure S29. T_2 Relaxation Data and Analysis for *apo*/*holo*-[Gd(DTPA-mal^N)]$_{72}$-GlnBP at 9.4 T........ S41
Figure S30. T_2 Relaxation Data and Analysis for *apo*/*holo*-[Gd(DOTA-mal^C)]$_{72}$-A$_{126}$Y-GlnBP at 9.4 T........ S42
Figure S31. T_1 Relaxation Data and Analysis for *apo*/*holo*-[Dy(DOTA-mal^N)]$_{72}$-GlnBP at 9.4 T S43
Figure S32. T_1 and T_2 Relaxation Data and Analysis for [Ln(DOTA)(H_2O)] at 9.4 T S44
Figure S33. T_2 Relaxation Data and Analysis for *apo*/*holo*-[Dy(DTPA-mal^N)]$_{72}$-G$_{119}$K-GlnBP at 9.4 T........ S45
Figure S34. T_2 Relaxation Data and Analysis for *apo*/*holo*-[Dy(DTPA-mal^N)]$_{72}$-A$_{126}$D-GlnBP at 9.4 T S46
Figure S35. T_2 Relaxation Data and Analysis for *apo*/*holo*-[Dy(DTPA-mal^N)]$_{72}$-N$_{127}$Y-GlnBP at 9.4 T........ S47
Figure S36. T_2 Relaxation Data and Analysis for *apo*/*holo*-[Gd(DTPA-mal^N)]$_{72}$-Q$_{183}$K-GlnBP at 9.4 T S48
Figure S37. T_2 Relaxation Data and Analysis for *apo*/*holo*-[Dy(DTPA-mal^N)]$_{72}$-Q$_{183}$Y-GlnBP at 9.4 T........ S49
Figure S38. Isomers Formed During Bioconjugation with DOTA-mal^N S50
Figure S39. Regioisomer model S51
Figure S40. Hydrogen-bond network in *holo*-[Gd(DOTA-mal^N)]$_{72}$-GlnBP S52
Figure S41. Gln electron density map in *holo*-[Gd(DOTA-mal^N)]$_{72}$-GlnBP S53
Figure S42. Hydrogen-bond network around Gln in *holo*-[Gd(DOTA-mal^N)]$_{72}$-GlnBP S54
Figure S43. Gd^{3+} ion electron density in *apo*-[Gd(DOTA-mal^N)]$_{72}$-GlnBP S55
Figure S44. Gd^{3+} ion electron density in *holo*-[Gd(DOTA-mal^N)]$_{72}$-GlnBP........ S56
Figure S45. Temperature-Dependent Relaxivities of *apo*- and *holo*-swArMs S57
References S58

I. Materials.

18.2 MΩ-cm resistivity water was obtained from a Milli-Q water purification system. D_2O was purchased from Cambridge Isotopes Laboratory. Tris HCl was purchased from Fisher Bioreagents. Guanidine HCl, (2-carboxyethyl)phosphine (TCEP), and Isopropylthio-β-d-galactoside (IPTG) were purchased from GoldBio. Glutamine, $[Gd(NO_3)_3]\bullet6H_2O$, $DyCl_3\bullet6H_2O$, 1,4,7,10-Tetraazacyclododecane-1,4,7,10-tetraacetic acid (DOTA), and Xylenol Orange tetrasodium salt were purchased from Millipore-Sigma. 1,4,7,10-Tetraazacyclododecane-1,4,7-tris-acetic acid-10-maleimidoethylacetamide (DOTA-mal^N) was purchased from Macrocyclics. 5-[2-(2,5-dioxopyrrol-1-yl)ethylamino]-5-oxo-2-[4,7,10-tris(carboxymethyl)-1,4,7,10-tetrazacyclododec-1-yl]pentanoic acid (DOTA-mal^C) was purchased from Smolecule. Ni-INDIGO agarose resin was purchased from Cube Biotech. Sephadex resin was purchased from Cytiva. TEV protease was expressed and purified according to literature protocols.[1] *E. coli* glutamine binding protein (GlnBP) and its variants were expressed and purified as previously published.[2]

Molecular Biology. Primers for all mutants are available in previous publications.[2,3]

swArM preparation. 1 M metal ion stock solutions were prepared in 1% nitric acid for short-term storage (up to 2 weeks at room temperature, RT). At the time of sample preparation, a 10-fold dilution into 0.1 M NaOAc buffer at pH 6.0 was performed. DOTA-mal^N stock solutions were prepared at 80 mM in DMSO, with the exact concentration determined by UV–vis absorption spectroscopy (ε_{302} = 620 M^{-1} cm^{-1}) and stored long term at –20 °C. To metalate, 90 µL of 80 mM DOTA-mal^N was mixed with 144 µL of 100 mM metal ion stock solution (2 eq.) and together diluted to a total volume of 1.8 mL with 0.1 M NaOAc at pH 6.0. Incubation at RT for 1 h with rocking afforded the metalated DOTA-mal^N in quantitative yield.

To ensure that single-cysteine variants of GlnBP (Cys-GlnBP) were fully reduced, 4 mL of 1.5 mM protein was incubated with 1 eq. of TCEP (from a 1M stock solution in water) for 1 h before addition of the above prepared solution (1.8 mL) followed by incubation for 2 h at RT with gentle rocking.

Excess metal was then removed by diluting three-fold in PBS buffer at pH 7.4 followed by centrifugation to remove solid precipitated unliganded metal ion. The supernatant of this solution was then concentrated by spin filtration to ~3 mL. Excess metalated DOTA-mal^N was then removed by size exclusion chromatography on a 12 x 2.6 cm (bed height x diameter) column packed with Sephadex G-25 fine resin (60 mL CV) and exchange into PBS buffer at pH 7.4. Fractions were pooled based on Bradford assay and then concentrated by spin filtration (10 kDa MWCO) to final volume of ~3 mL at ~1.5 mM.

swArMs produced by the above protocol were characterized by LC-MS (Figures S1-S5), where the expected mass shift for bioconjugation of [M(DOTA-mal^N)] was observed within one or two protons ([Gd(DOTA-mal^N)]: 682 Da, [Dy(DOTA-mal^N)]: 686 Da). Minimal oxidized protein (which could form by irreversible Cys oxidation), no excess metal ions, and

no bound unmetalated DOTA-mal^N were observed using the above protocols. DPTA-mal^N and NOTA-mal^N swArMs were prepared identically to DOTA-mal^N swArMs.

(Ln)DOTA Preparation. DOTA and metal stocks were prepared at 20 mM in Milli-Q H_2O and combined in a 1:1 ratio. The solution was adjusted to pH 7-8 with small amounts of concentrated NaOH and allowed to equilibrate for 1 hour at 37 °C. A xylenol orange assay was used to detect the presence of free metal by adding 25 μL of the resultant Ln(DOTA) solution to 475 μL of 10 μM xylenol orange in 50 mM NaOAc buffer at pH 5.8. If free metal was detected, 0.1 equivalents of DOTA were added, followed by pH adjustment, equilibration, and retesting. This was repeated until free metal was no longer detected. The solution was combined in a 1:1 ratio with 2x PBS buffer at pH 7.4, and the final concentration of metal was determined by EDXRF (see next section).

II. Physical Methods and Instrumentation

Energy-Dispersive X-Ray Fluorescence (EDXRF) Spectroscopy. Metal concentrations were determined using a Shimadzu EDX-7200 instrument. 300 μL solutions were placed in sample cups and covered in 6 μm Mylar film. Data were collected for 200 s under a He atmosphere. Intensity was calculated by integrating under the peak of the Lα line (Gd: 6.053 keV, Dy: 6.498 keV). A calibration curve for each metal was generated using 5 concentrations spanning 0.5 mM – 2.5 mM, and protein samples were prepared at 3 concentrations for comparison. An exemplary calibration curve is provided in Figure S6.

Protein Quantification. Protein was quantified by the previously measured extinction coefficient of $\varepsilon_{280} = 25.9\ mM^{-1}\ cm^{-1}$.

Isothermal Titration Calorimetry (ITC). The affinities of GlnBP variants and swArM constructs for Gln were determined by ITC using a TA Instruments Affinity instrument and recording via Nano ITC software, with analyses performed using NanoAnalyze. For each experiment, 50 μM protein samples in PBS buffer at pH 7.4 were added to the sample cell, and 320 μM Gln samples in matched buffer added to the syringe. The system was equilibrated to 37° C, and 20–30 2.5 μL injections were performed. The data were fit to an independent binding model plus a linear function to account for background heat released.

Circular Dichroism (CD) Spectroscopy. CD spectra were recorded using a Jasco J-1500 spectrophotometer. Protein samples were prepared at 50 μM in PBS pH 7.4, in the presence of 10x Gln for *holo*-samples. Spectra were recorded at 37 °C. A blank spectrum of PBS was taken first and used as a baseline for all measurements.

Protein Crystallography, Data Collection, and Structural Solution

A. **Sample preparation.** Single crystal samples of *apo*-$[Gd(DOTA)]_{72}$-swArMs were grown using previously published conditions.[3] Single crystal samples of *holo*-$[Gd(DOTA)]_{72}$-swArMs were grown with the same method, but with the crystallization buffer consisting of 0.17 M $(NH_4)_2SO_4$, 0.1 M NaOAc, 25 % W/V PEG-MME 2000, pH 4.6, and the

cryopreservation buffer consisting of 0.2 M $(NH_4)_2SO_4$, 0.1 M NaOAc, 25 % W/V PEG-MME 2000, 20% V/V glycerol, pH 4.6.

B. X-ray Data Collection and Processing. Both the *apo* and *holo* datasets of swArM were collected on separate dates using the Stanford Synchrotron Radiation Lightsource (SSRL, SLAC National Accelerator Laboratory) 12-2 beamline (Dectris Eiger2 XE 16M pixel array detector).[4–7] Both datasets were collected at 0.97949 Å while the crystals were immersed in a cryostream at a temperature of 100 K. The wavelength of 0.97949 Å was chosen for collection because it is the K edge for Se, which is a high energy incident beam allowing for high-resolution data collection and also allows for significant anomalous signal to be observed for elements in the lanthanide series, such as Gd^{3+}, which is present in both structures.

Both datasets were processed using XDS[8,9] for indexing, integration, and scaling. For the *apo*-structure, a resolution cut-off of 2.02 Å was used due to a significant increase in R_{meas} in data at higher resolutions, even though the I/σ value for data at a resolution of 2.02 Å still measures 2.08. The *apo* dataset was indexed as space group P 6_3 2 2 with unit cell 128.16 128.16 119.83 90.00 90.00 120.00. For the *holo*-structure, many different crystals were screened due to a challenging plate-like morphology. One dataset, however proved sufficient for structure solution. The *holo* dataset has a resolution cut-off of 2.57 Å, with an I/σ value of 1.94. The space group for the *holo* dataset is P 6_5 with a unit cell of 221.44 221.44 79.56 90.00 90.00 120.00.

C. Phasing and Structural Refinement. Phases for both datasets were obtained using Molecular Replacement in Phenix[10,11] with a search model of a previously published glutamine-bound (8EYZ) glutamine binding protein structure for the *apo*- and *holo*-structures.[2] For the *apo* dataset, the search model was split into two lobes to accommodate for the structural change that occurs when glutamine is not present. In both datasets, molecular replacement produced high phasing statistics indicating plausible solutions were found. For the *apo* dataset, a LLG of 2175.93 and a TFZ of 48.4 was obtained whereas for the *holo* dataset, a LLG of 13058.64 and a TFZ 77.9 was obtained. Once phases were obtained for both datasets, starting model bias was removed using 10 cycles of simulated annealing. The *apo* dataset contains one molecule per asymmetric unit with an estimated solvent content of 0.777 and adopted the open conformation, as expected in the absence of glutamine.[12] The *holo* dataset contains six molecules per asymmetric unit with an estimated solvent content of 0.663. In this structure, each monomer exhibits a closed conformation, with each monomer bound to a single molecule of glutamine.

5-percent of reflections from each dataset were used as a test set for structural refinement, which was accomplished using COOT and Phenix.[13,14] In both models, each chain shows a significant area of electron density near C72, which is the attachment point for the Gd-DOTA ligand of interest. Furthermore, when the data is processed anomalously, the observed pockets of electron density exhibit anomalous electron density, indicating that they are a result of the Gd-DOTA ligand bound to the protein. In both structures, the Gd-DOTA ligand itself was modeled in the Electronic Ligand Builder and Optimization Workbench (eLBOW)[15] without the presence of Gd^{3+}, which was added separately. Initial construction of the DOTA ligand in eLBOW had incorrect geometry, so a starting model from a published structure of Gd-DOTA (CSD ID: JOPJIH01) was used in conjunction with manual addition of the post ring-opening tail portion of the maleimide linker. As previously observed,[16] crystallographic programs misidentify Gd^{3+} (REFMAC ID: GD) as guanosine (REFMAC ID: Gd), and to overcome this error a single atom of Gd^{3+} was created using eLBOW. To account for the two potential isomeric attachment points of the DOTA ligand to the target C72 depending on maleimide ring-opening, in both the *apo*- and *holo*- structures, each monomer has both a split DOTA ligand and split Gd^{3+} ion. In both structures, the DOTA ligand and Gd^{3+} ions have high B-factors relative to the B-factors of the rest of the structure. These B-factors indicate highly flexible DOTA ligands, and this observation correlates with the solvent-rich locations of the ligands in each structure. Due to this flexibility, the anomalous maps were primarily used to accurately guide placement of the ligands.

Once each model for both the *apo*- and *holo*-structures were refined, model quality, geometry, and completeness were evaluated through the use of MolProbity[17]. In both final structures, there are no Ramachandran outliers; in the *apo*-structure 99-percent of residues adopt a favored position and 1-percent of residues adopt an allowed position whereas in the *holo*-structure 98-percent of residues adopt a favored position and 1-percent of residues adopt an allowed position. Both structures have 100-percent rotameric sidechains, with no outliers. In the *apo*-structure, the first 5 N-terminal residues are not modeled due to no supporting density. In the *holo*-structure the first 5 residues in chains B, C, D, and E are not modeled due to no supporting density, and in chain F the first seven residues are not modeled due to no supporting density. In chains B and D, the final C-terminal lysine residue is not modeled due to no supporting density, and in chains E and F the final 4 C-terminal residues are not modeled due to no supporting density. Final model quality was judged using simulated annealing composite omit electron density maps. Figures showing protein structures were made using PyMOL[18] and the crystallography software packages used to solve the structures were compiled by SBGrid.[19]

Nuclear Magnetic Resonance (NMR) Spectroscopy and Relaxivity Measurements

A. **Sample preparation.** Relaxivity was determined by measuring the T_1 and T_2 relaxation times for a range of sample concentrations in PBS buffer, at pH 7.4 and 37 °C containing

10 % D_2O (assay buffer). Stock solutions of swArM in assay buffer was prepared at 1.11, 0.83, 0.56, and 0.28 mM. *Apo*- and *holo*-swArM samples were prepared from the same stock by adding either 20 µL assay buffer or 20 µL 0.1 M Gln in assay buffer, to 180 µL of protein solution, respectively. Final protein concentrations were verified using the molar extinction coefficient ε_{280} = 25.9 mM^{-1} cm^{-1}.

B. **NMR Measurements.** Relaxivities of all variants were determined at 37 °C from spectra taken at 9.4 T (400 mHz) using a Varian Inova 400-MHz NMR spectrometer equipped with a Nalorac, 5 mm, DR probe. Select variants were also investigated at 32 °C at 1.4 T (60 mHz) using a Nanalysis NMReady60 Pro instrument. Measurements were typically performed on 4 different concentrations of contrast agent and on the same buffer with no contrast agent. Temperature-dependent relaxivity measurements were performed using a Varian Inova 500-MHz NMR spectrometer, equipped with a Varian, 5-mm, TR PFG probe with inverse proton detection. Temperature-dependent relaxivity was determined using either two concentrations of contrast agent (0.5 and 1.0 mM) or three (0.3, 0.6, and 0.9 mM). The temperature was calibrated using the ^{1}H NMR signal of ethylene glycol.[20]

T_1 measurements were performed using the inversion recovery method[21] and T_2 measurements were performed using the Carr–Purcell–Meiboom–Gill (CPMG) pulse sequence[22].

Six or more pulse delay times (τ) were chosen to ensure coverage of the T_i under observation, and the delay between pulse sequences was set to at least 5-times T_1. Optimized 90° and 180° pulse widths were determined on the day of the experiment, and typically spanned 16–20 µs (90° pulse width) and 32–40 µs (180° pulse width). The delay in the CPMG pulse sequence was set to at least 50 times the 180° pulse width to allow at least four echo loops.

C. **Analysis.** To determine T_i, data were analyzed via MestReNova software. For each spectrum, an integration was performed over the water peak, and the values obtained (dependent variable) were plotted against τ (independent variable). These data were then fit to a monoexponential decay according to Eq. 1. From these analyses, we obtain T_i for each sample concentration and type.

$$y = y_o + Ae^{\frac{\tau}{T_i}} \tag{1}$$

$$\frac{1}{T_i} - \frac{1}{T_i^0} \tag{2}$$

The quantity described by Eq. 2, where T_i at zero added sample is T^0, was then plotted against the swArM concentration and a linear regression model was applied. The slope of this line gives the contrast agent relaxivity r_i.

Table S1: K_d values, $\Delta H°$, and $\Delta S°$ of Gln Dissociation from GlnBP variants.[a]

swArM or GlnBP Variant	K_d (μM)	n	ΔH (kJ/mol)	ΔS (J/mol·K)
$T_{72}C$	0.6 ± 0.1	0.93 ± 0.01	-52.1 ± 0.3	-48 ± 3
$[Gd(DOTA\text{-}mal^N)]_{72}$	1.8 ± 0.8	0.9 ± 0.1	-44 ± 2	-30 ± 10
$[Gd(DTPA\text{-}mal^N)]_{72}$	1. 6	1.0	−46	−37
$[Gd(DOTA\text{-}mal^N)]_{122}$	1.1	0.89	−66	−100
$N_{160}C$	0.11	0.89	−60	−60
$[Gd(DOTA\text{-}mal^N)]_{160}$	810	0.56	−99	−260
$T_{72}C\text{-}G_{119}R$	1.1	1.0	−43	−30
$[Dy(DOTA\text{-}mal^N)]_{72}\text{-}G_{119}R$	1.8	0.73	−10	79
$T_{72}C\text{-}A_{126}D$	1.4	1.1	−53	−66
$[Dy(DOTA\text{-}mal^N)]_{72}\text{-}A_{126}D$	1.2	0.97	−41	−18
$T_{72}C\text{-}A_{126}Y$	0.7	1.0	-51	-47
$[Dy(DOTA\text{-}mal^N)]_{72}\text{-}A_{126}Y$	2.9	0.89	-52	-63
$[Dy(DOTA\text{-}mal^N)]_{72}\text{-}N_{127}Y$	1.2	0.99	−38	−8.2
$T_{72}C\text{-}Q_{183}Y$	2.0	0.85	−43	−34
$[Dy(DOTA\text{-}mal^N)]_{72}\text{-}Q_{183}Y$	2.8	0.89	−36	−9.8

[a]Assessed by isothermal titration calorimetry (ITC) from fits to an independent binding model plus a linear function to account for background heat released. Uncertainty limits correspond to 1 s.d. from duplicate measurements, otherwise measurements were only performed once. All measurements were collected for samples in matched PBS buffer, pH 7.4, 37 °C.

Table S2: Single-Mutant Relaxivity Values (mM^{-1} s^{-1}) Assessed at 1.4 T.[a]

swArM	r_1 (Apo)	r_1 (Holo)	Holo/Apo (%)	r_2 (Apo)	r_2 (Holo)	Holo/Apo (%)
$[Gd(DOTA\text{-}mal^N)]_{72}$[b]	19.5 ± 0.4	20.4 ± 0.3	4 ± 2	37.2 ± 0.7	41.5 ± 0.4	11 ± 2
$[Gd(DTPA\text{-}mal^N)]_{72}$[c]	16.7 ± 0.6	17.8 ± 0.2	7 ± 4	27 ± 1	30.3 ± 0.9	11 ± 6
$[Dy(DOTA\text{-}mal^N)]_{72}$[c]	0.176 ± 0.007	0.201 ± 0.001	14 ± 5	0.63 ± 0.05	0.87 ± 0.06	40 ± 20
$[Gd(DOTA\text{-}mal^N)]_{122}$[c]	11.5 ± 0.2	12.6 ± 0.3	10 ± 4	17.3 ± 0.4	19.3 ± 0.3	12 ± 3
$[Dy(DOTA\text{-}mal^N)]_{122}$[c]	0.346 ± 0.008	0.37 ± 0.03	10 ± 10	0.7 ± 0.1	0.62 ± 0.04	0 ± 20

[a]Uncertainty limits in r_i represent the standard error of the fit. Uncertainty limits on the percentage change in *holo*/*apo* relaxivity are from propagation of uncertainty in each individual value (*apo* and *holo*). [b]Determined from two sets of independently prepared samples. [c]Determined from one set of independently prepared samples. Samples were prepared in PBS buffer, pH 7.4, and measurements were taken at 32 °C.

Table S3: Single-Mutant Relaxivity Values (mM^{-1} s^{-1}) Assessed at 9.4 T.[a]

swArM	r_1 (Apo)	r_1 (Holo)	Holo/Apo (%)	r_2 (Apo)	r_2 (Holo)	Holo/Apo (%)
$[Gd(DOTA\text{-}mal^{N})]_{72}$[b]	4.35 ± 0.07	4.32 ± 0.07	−1 ± 2	36 ± 1	43.2 ± 0.8	20 ± 5
$[Gd(DTPA\text{-}mal^{N})]_{72}$[c]	4.0 ± 0.1	4.14 ± 0.05	3 ± 3	65 ± 4	72 ± 4	10 ± 10
$[Dy(DOTA\text{-}mal^{N})]_{72}$	0.37 ± 0.01[c]	0.37 ± 0.02[c]	−1 ± 5[c]	6.86 ± 0.08[d]	9.3 ± 0.1[d]	36 ± 3[d]
$[Gd(DOTA\text{-}mal^{N})]_{122}$[c]	4.00 ± 0.03	4.04 ± 0.03	1 ± 1	17.97 ± 0.08	19.6 ± 0.3	9 ± 2
$[Dy(DOTA\text{-}mal^{N})]_{122}$[c]	0.359 ± 0.006	0.37 ± 0.01	2 ± 3	10 ± 1	11 ± 1	1.0 ± 0.2
$[Gd(DOTA\text{-}mal^{N})]_{160}$[c]	3.3 ± 0.1	3.49 ± 0.06	5 ± 5	80 ± 4	73 ± 4	−9 ± 6

[a]Uncertainty limits in r_i represent the standard error of the fit. Uncertainty limits on the percentage change in *holo*/*apo* relaxivity are from propagation of uncertainty in each individual value (*apo* and *holo*). [b]Determined from three sets of independently prepared samples. [c]Determined from one set of independently prepared samples. [d]Determined from two sets of independently prepared samples. Samples were prepared in PBS buffer, pH 7.4, and measurements were taken at 37 °C.

Table S4: Double-Mutant Relaxivity Values (mM^{-1} s^{-1}) Assessed at 9.4 T.[a]

swArM	r_2 (Apo)	r_2 (Holo)	Holo/Apo (%)
$[Dy(DOTA\text{-}mal^{N})]_{72}$-$G_{119}K$[b]	7.39 ± 0.07	8.5 ± 0.2	15 ± 4
$[Dy(DOTA\text{-}mal^{N})]_{72}$-$A_{126}D$[b]	9.5 ± 0.3	11.7 ± 0.09	23 ± 5
$[Dy(DOTA\text{-}mal^{N})]_{72}$-$A_{126}Y$[c]	6.8 ± 0.2	10.8 ± 0.2	58 ± 6
$[Dy(DOTA\text{-}mal^{C})]_{72}$-$A_{126}Y$[b]	9.7 ± 0.1	9.6 ± 0.2	−1 ± 2
$[Dy(DOTA\text{-}mal^{N})]_{72}$-$N_{127}Y$[c]	6.5 ± 0.3	9.1 ± 0.2	40 ± 7
$[Gd(DOTA\text{-}mal^{N})]_{72}$-$Q_{183}K$[b]	30.9 ± 0.2	28.2 ± 0.8	−8 ± 2
$[Dy(DOTA\text{-}mal^{N})]_{72}$-$Q_{183}Y$[b]	14.0 ± 0.1	18.0 ± 0.4	29 ± 4

[a]Uncertainty limits in r_i represent the standard error of the fit. Uncertainty limits on the percentage change in *holo*/*apo* relaxivity are from propagation of uncertainty in each individual value (*apo* and *holo*). [b]Determined from one set of independently prepared samples. [c]Determined from two sets of independently prepared samples. Samples were prepared in PBS buffer, pH 7.4, and measurements were taken at 37 °C.

Table S5. XRD Processing and Refinement Statistics for [Gd(DOTA-mal^N)]-GlnBP.

	Holo-[Gd(DOTA-malN)]$_{72}$-GlnBP[a]	*Apo*-[Gd(DOTA-malN)]$_{72}$-GlnBP[b]
**Data collection**		
Space group	$P6_5$	$P6_3 2 2$
Cell dimensions		
a, b, c (Å)	221.44 221.44 79.56	128.16 128.16 119.83
α, β, γ (°)	90, 90, 120	90 90 120
Resolution (Å)	40.00 – 2.57 (2.66 – 2.57)	41.95 – 2.02 (2.09 – 2.02)
R_{meas} (%)	12.40 (93.40)	6.10 (120.2)
I / σ	14.79 (1.94)	23.81 (2.08)
Completeness (%)	99.60 (99.02)	99.2 (98.6)
Redundancy	9.60 (8.82)	13.19 (13.75)
CC1/2	99.9 (88.6)	100 (80.2)
**Refinement**		
Resolution (Å)	37.44 – 2.57	35.35 – 2.02
Unique reflections	70744 (6979)	38279 (3743)
R_{work} / R_{free}	**0.2074 / 0.2365**	**0.1907 / 0.2263**
No. atoms	11500	2219
Protein	10497	1814
Gd	12[a]	2[b]
DOTA	456[a]	76[b]
Glutamine	60	–
SO_4	30	20
Glycerol	54	6
Ethylene glycol	–	20
Acetate	40	12
Solvent	351	269
**B-factors**		
Overall	61.03	56.96
Protein	57.80	51.65
Gd	204.15	198.04
DOTA	127.42	132.59
Glutamine	50.35	–
SO_4	88.52	73.17
Glycerol	75.06	77.25
Ethylene glycol	–	78.23
Acetate	77.81	79.62
Solvent	61.71	66.13
**R.M.S. deviations**		
Bond lengths (Å)	0.011	.007
Bond angles (°)	1.60	.83

[a]There are six molecules per ASU, each molecule has a single DOTA and Gd ion bound, but each DOTA and Gd adopt a split confirmation based on attachment isomers.
[b]There is one molecule of DOTA and one Gd ion, but each adopts a split confirmation based on attachment isomers.

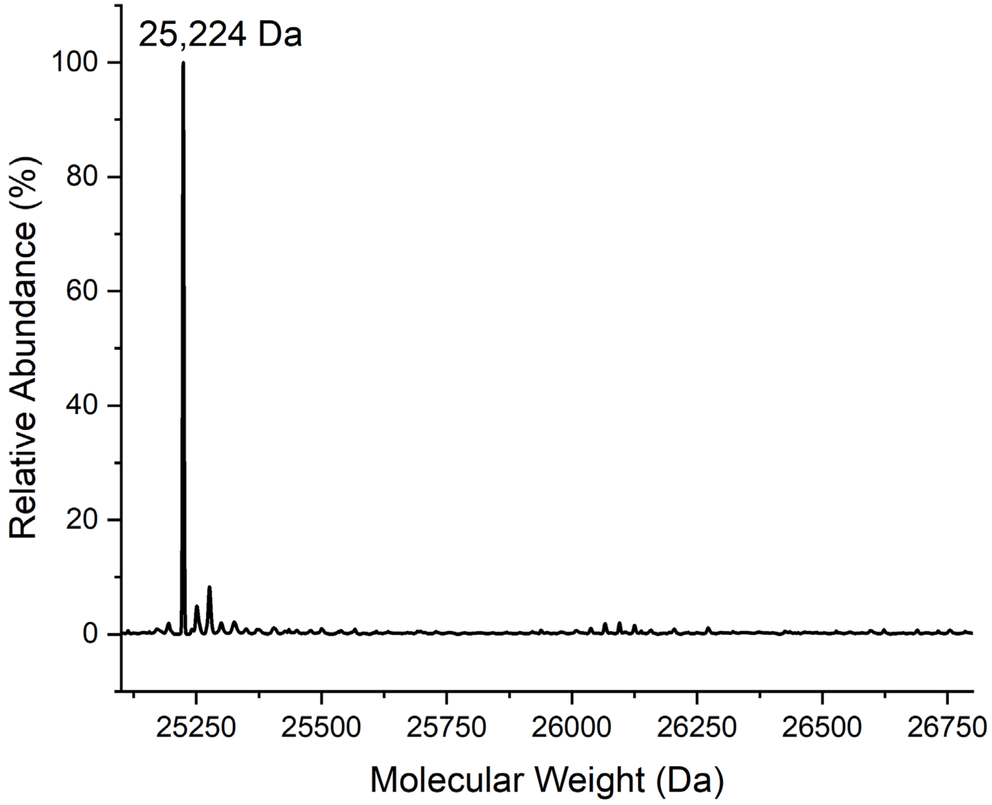

Figure S1. Mass spectrum of T_{72}C-GlnBP from LC-MS analysis.

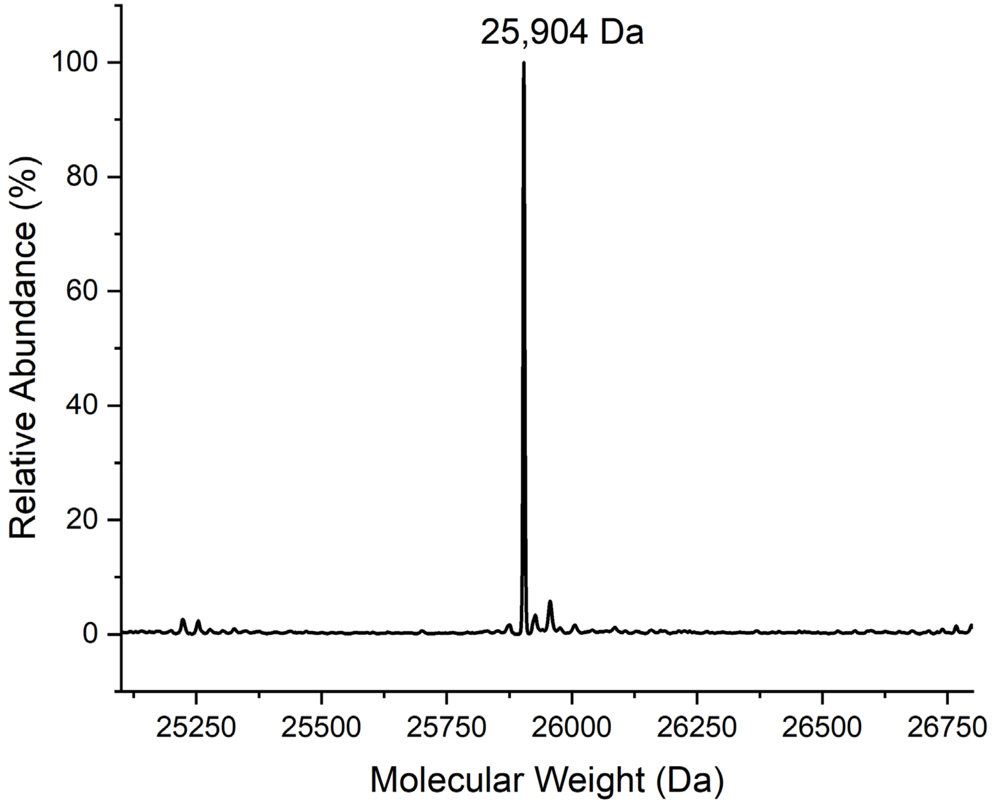

Figure S2. Mass spectrum of [Gd(DOTA-mal^{N})]$_{72}$-GlnBP from LC-MS analysis. The two minor peaks on the left correspond to residual unconjugated GlnBP, separated by ~31 Da. The left peak matches the mass of reduced T$_{72}$C-GlnBP, while the right peak matches the mass of doubly-oxidized T$_{72}$C-GlnBP.

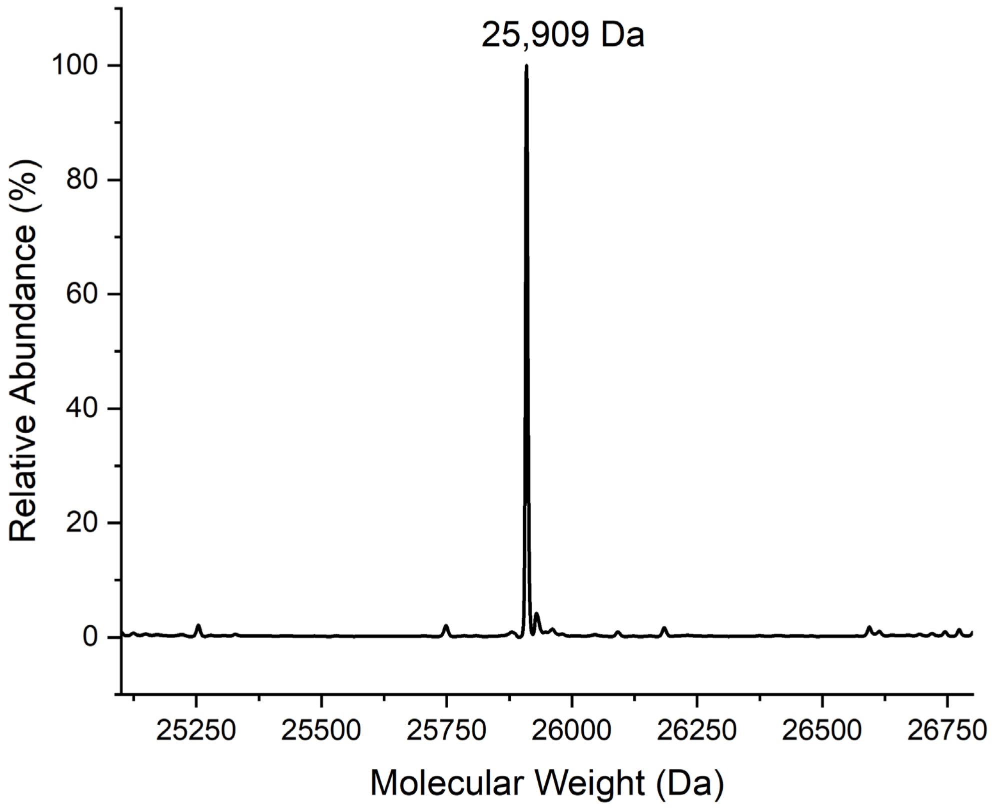

Figure S3. Mass spectrum of $[Dy(DOTA\text{-}mal^{N})]_{72}$-GlnBP from LC-MS analysis.

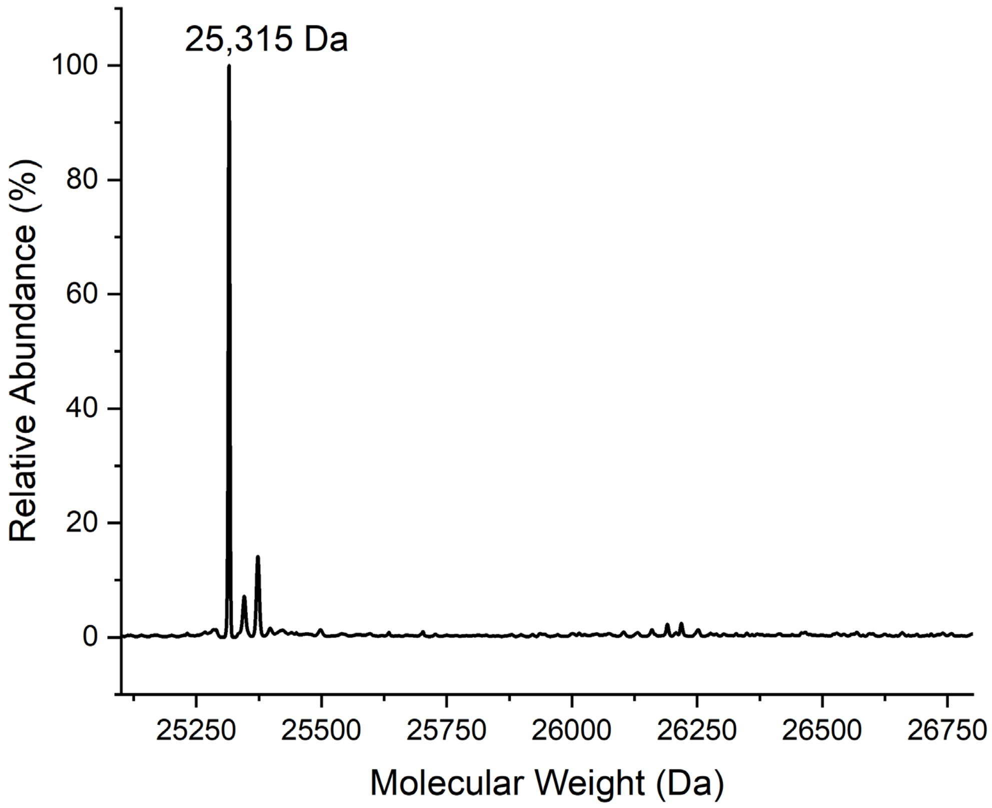

Figure S4. Mass spectrum of $T_{72}C$-$A_{126}Y$-GlnBP from LC-MS analysis.

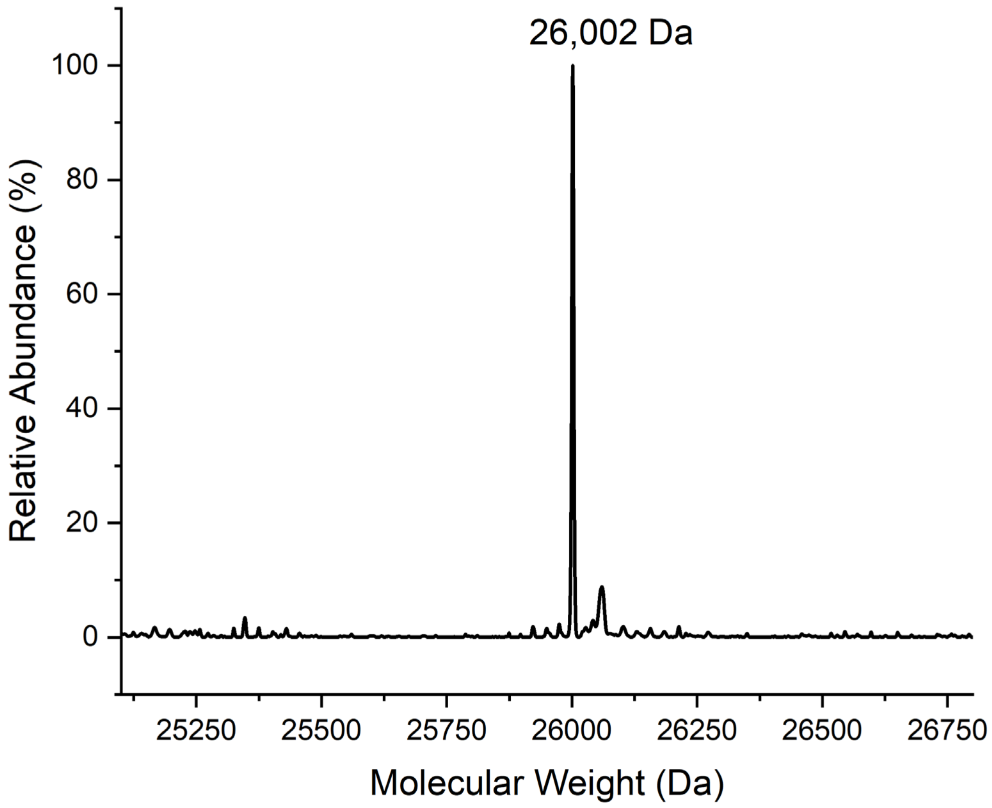

Figure S5. Mass spectrum of [Dy(DOTA-mal^{N})]$_{72}$-A$_{126}$Y-GlnBP from LC-MS analysis.

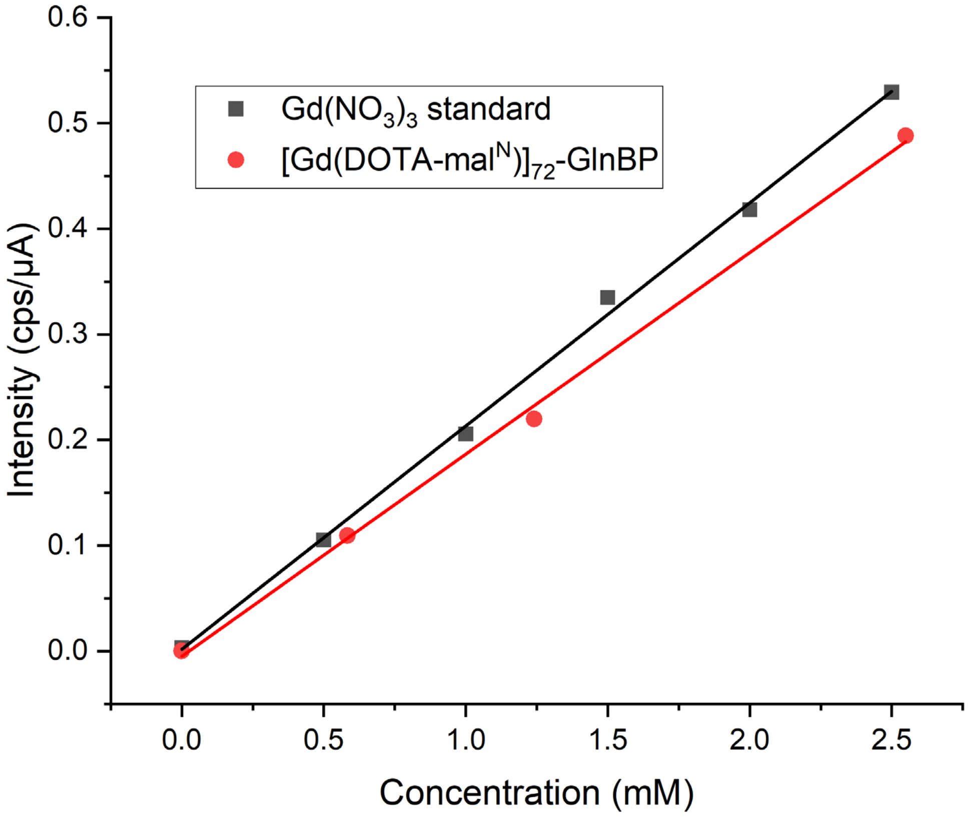

Figure S6: Calibration curve for EDXRF quantification of metal content of swArMs. EDXRF measurements (•,•) and linear fit (—,—) for $Gd(NO_3)_3$ standard and [Gd(DOTA-mal^N)]$_{72}$-GlnBP, respectively. Concentration of the standard was calculated by dilution from a 1 M stock, and concentration of the protein was determined via UV–vis absorption spectroscopy.

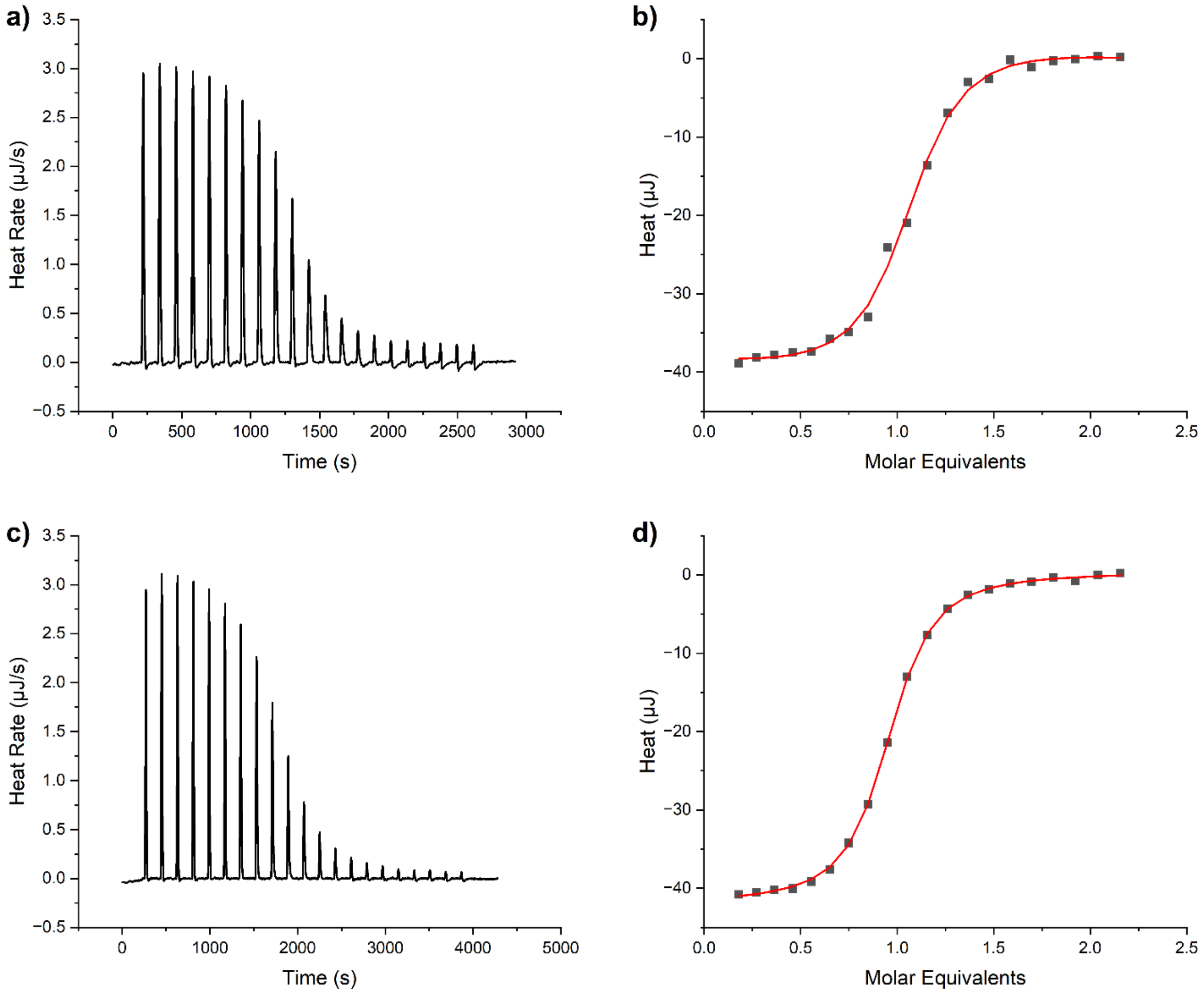

Figure S7. Isothermal titration calorimetry thermogram for addition of Gln to T72C-GlnBP (a, c) and integrated heat per injection (•), fit using NanoAnalyze with an independent binding model plus a linear function to account for background heat released (—) (b, d). Top and bottom panels are separate replicates.

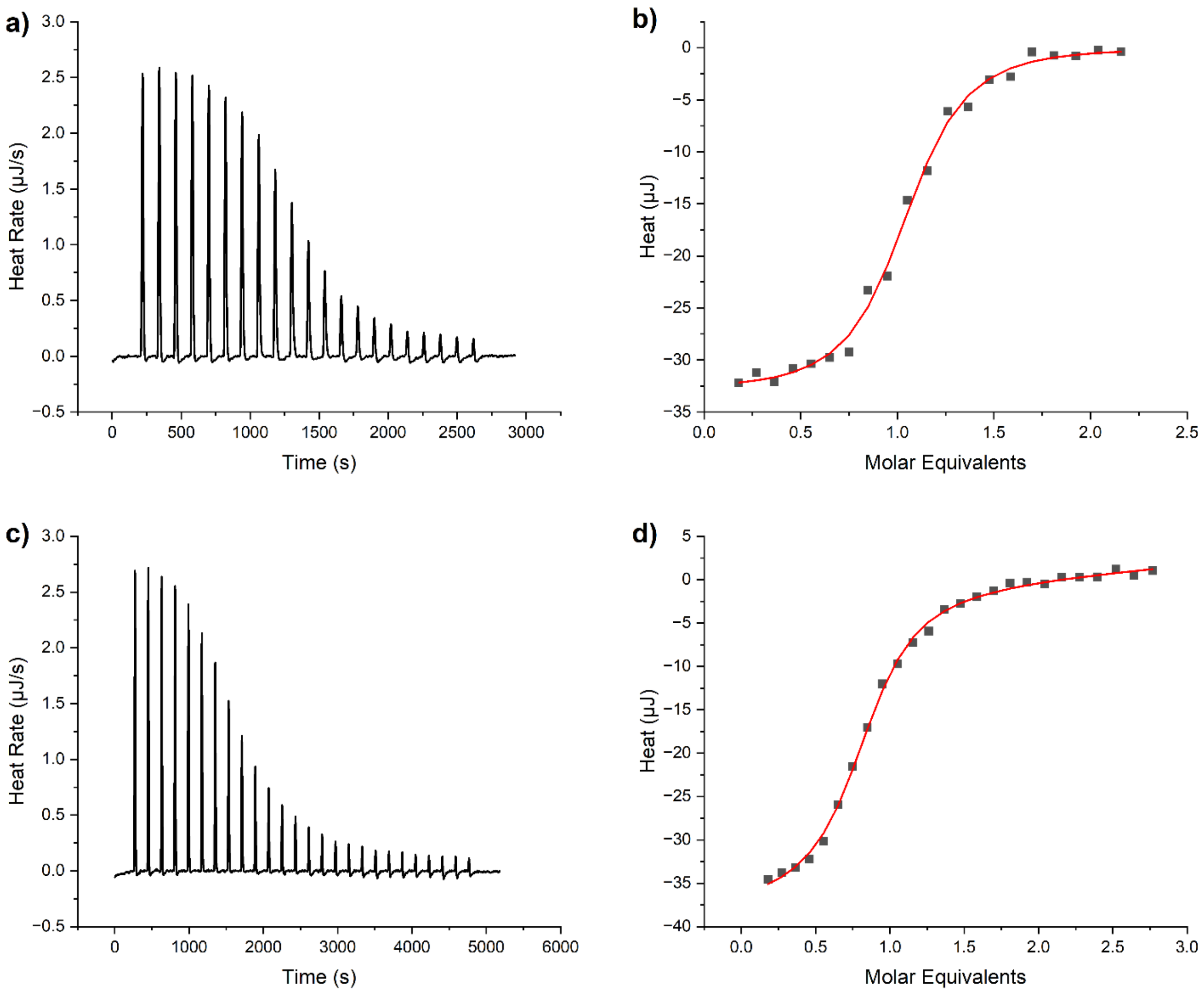

Figure S8. Isothermal titration calorimetry thermogram for addition of Gln to [Gd(DOTA-mal^{N})]$_{72}$-GlnBP (a, c) and integrated heat per injection (•), fit using NanoAnalyze with an independent binding model plus a linear function to account for background heat released (—) (b, d). Top and bottom panels are separate replicates.

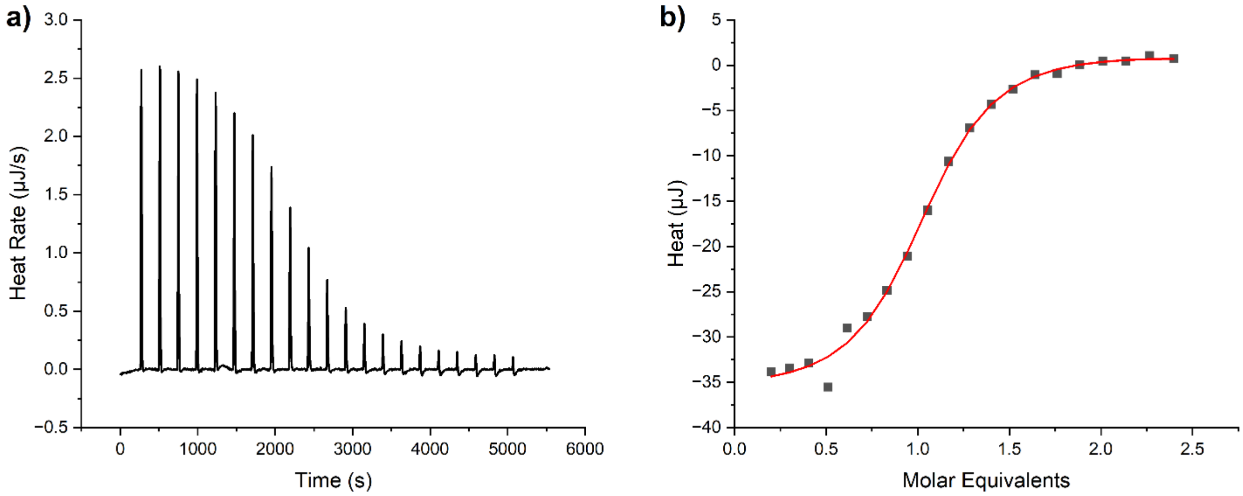

Figure S9. Isothermal titration calorimetry thermogram for addition of Gln to [Gd(DTPA-mal^{N})]$_{72}$-GlnBP (a) and integrated heat per injection (•), fit using NanoAnalyze with an independent binding model plus a linear function to account for background heat released (—) (b).

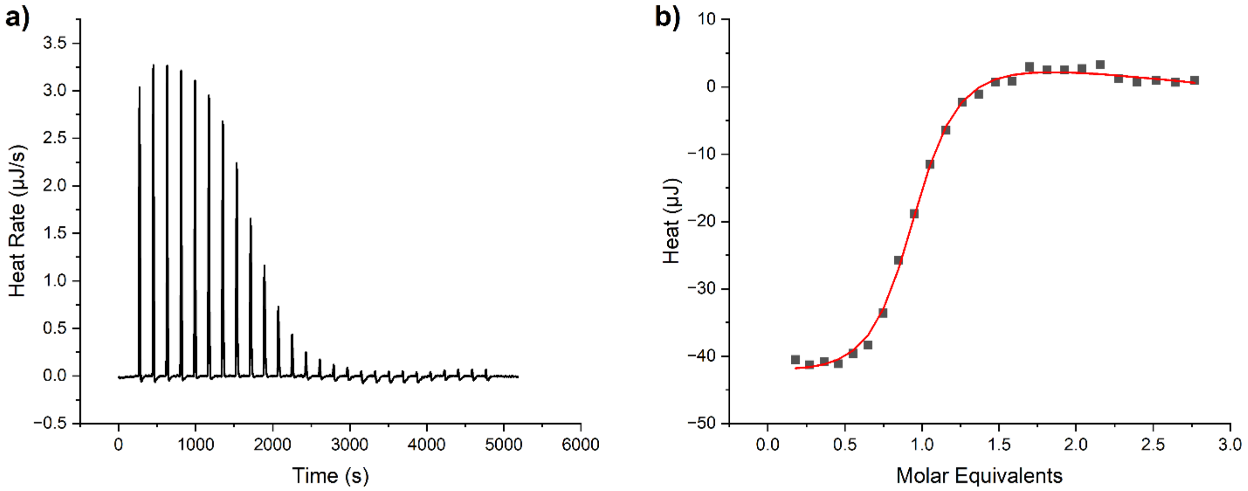

Figure S10. Isothermal titration calorimetry thermogram for addition of Gln to [Gd(DOTA-mal^{N})]$_{122}$-GlnBP (a) and integrated heat per injection (•), fit using NanoAnalyze with an independent binding model plus a linear function to account for background heat released (—) (b).

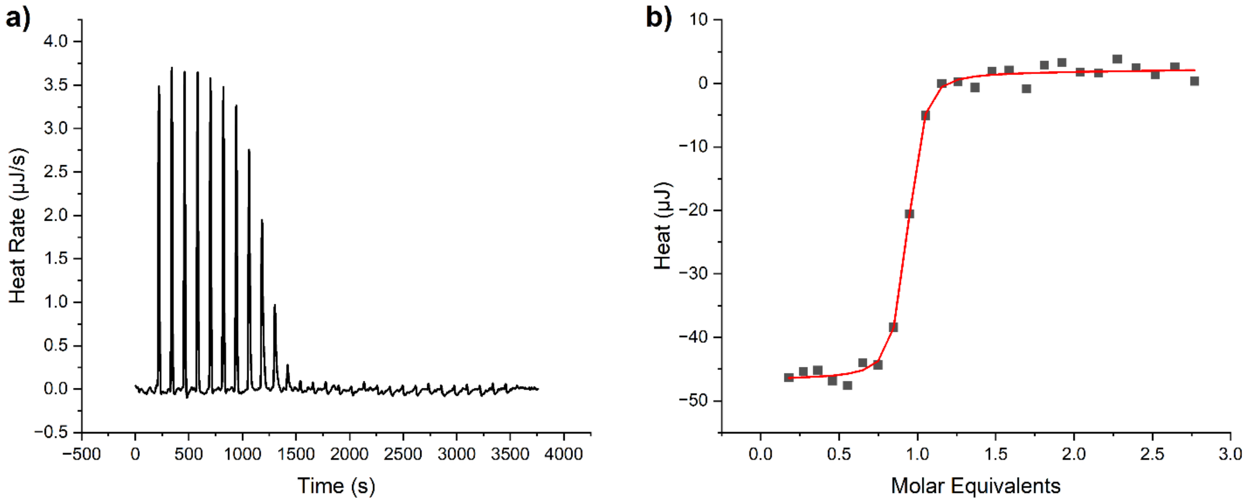

Figure S11. Isothermal titration calorimetry thermogram for addition of Gln to $N_{160}C$-GlnBP (a) and integrated heat per injection (•), fit using NanoAnalyze with an independent binding model plus a linear function to account for background heat released (—) (b).

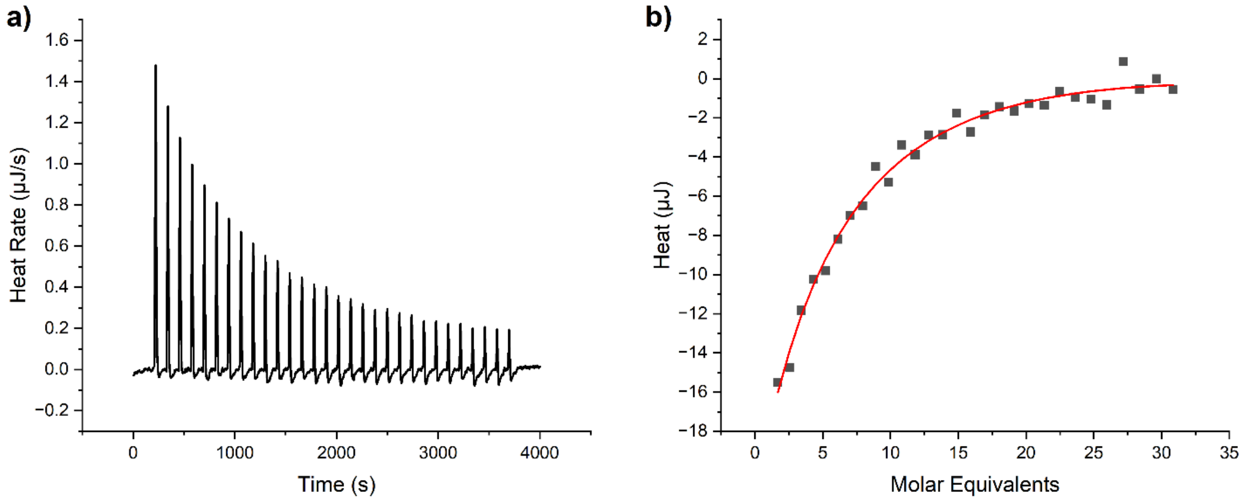

Figure S12. Isothermal titration calorimetry thermogram for addition of Gln to [Gd(DOTA-mal^{N})]$_{160}$-GlnBP (a) and integrated heat per injection (•), fit using NanoAnalyze with an independent binding model plus a linear function to account for background heat released (—) (b).

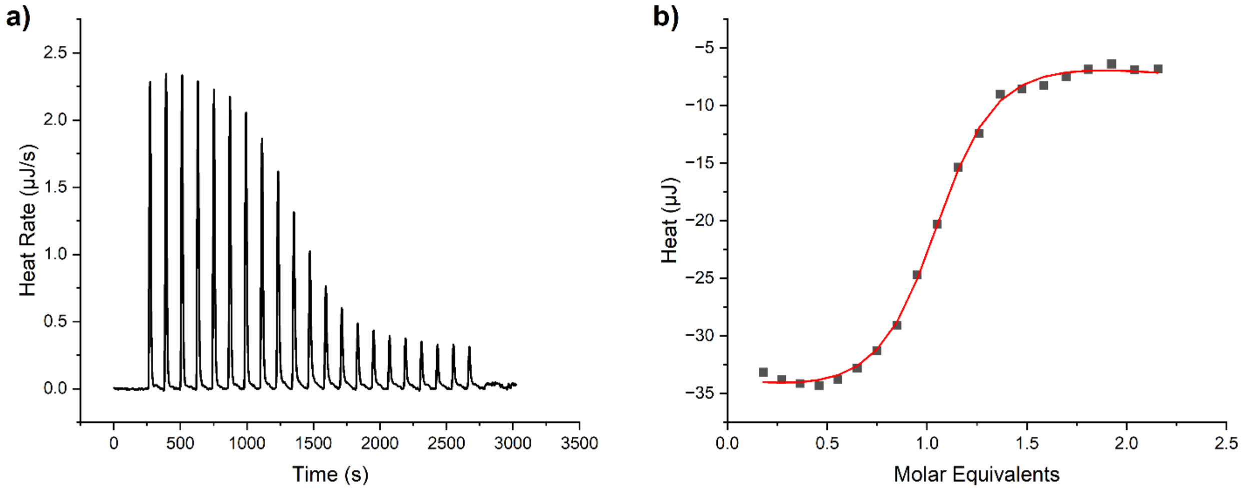

Figure S13. Isothermal titration calorimetry thermogram for addition of Gln to $T_{72}C$-$G_{119}R$-GlnBP (a) and integrated heat per injection (•), fit using NanoAnalyze with an independent binding model plus a linear function to account for background heat released (—) (b).

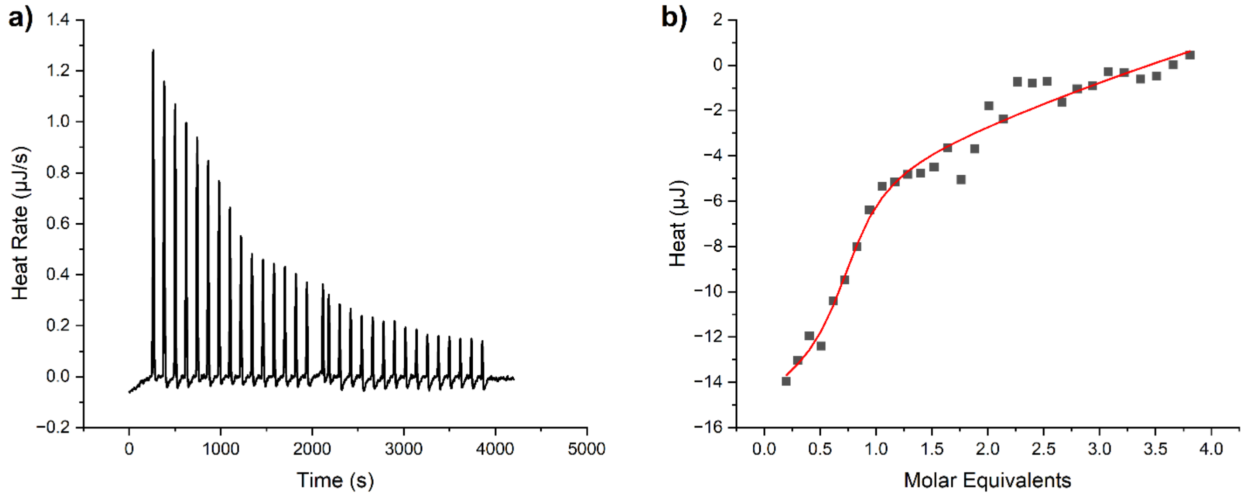

Figure S14. Isothermal titration calorimetry thermogram for addition of Gln to [Dy(DOTA-mal^{N})]$_{72}$-G$_{119}$R-GlnBP (a) and integrated heat per injection (•), fit using NanoAnalyze with an independent binding model plus a linear function to account for background heat released (—) (b).

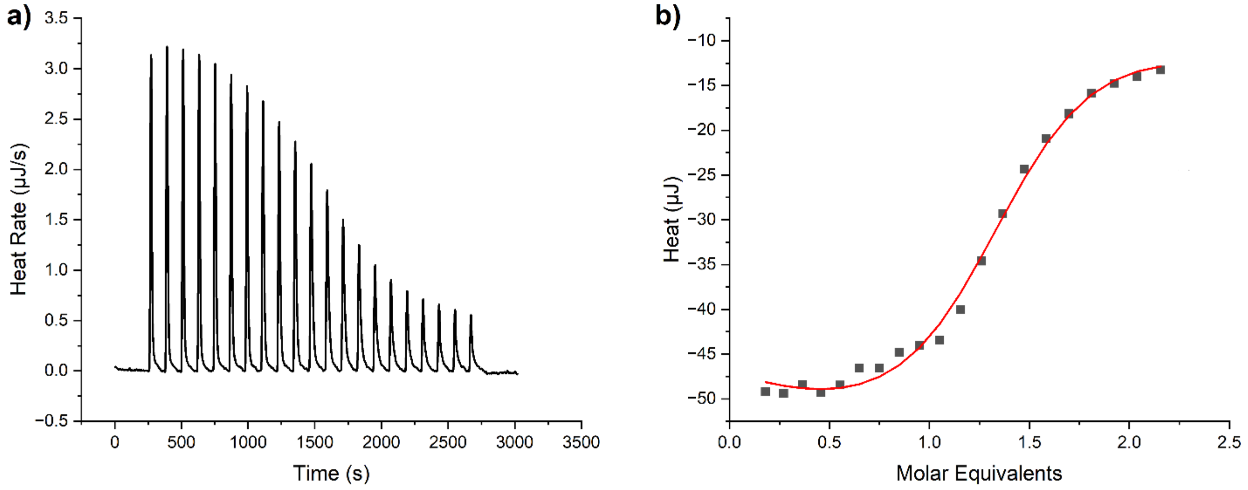

Figure S15. Isothermal titration calorimetry thermogram for addition of Gln to $T_{72}C$-$A_{126}D$-GlnBP (a) and integrated heat per injection (•), fit using NanoAnalyze with an independent binding model plus a linear function to account for background heat released (—) (b).

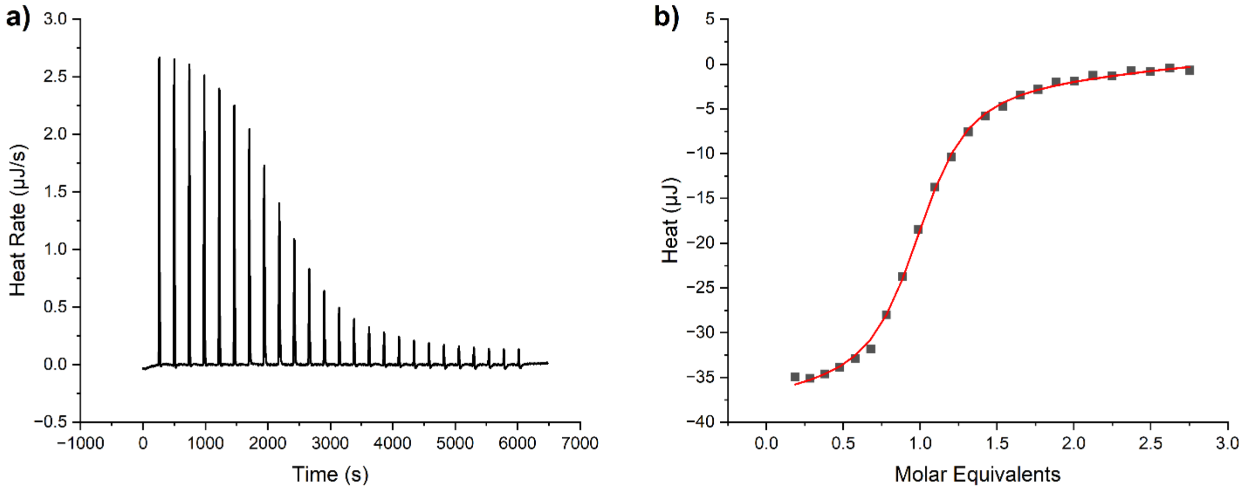

Figure S16. Isothermal titration calorimetry thermogram for addition of Gln to [Dy(DOTA-mal^{N})]$_{72}$-A$_{126}$D-GlnBP (a) and integrated heat per injection (•), fit using NanoAnalyze with an independent binding model plus a linear function to account for background heat released (—) (b).

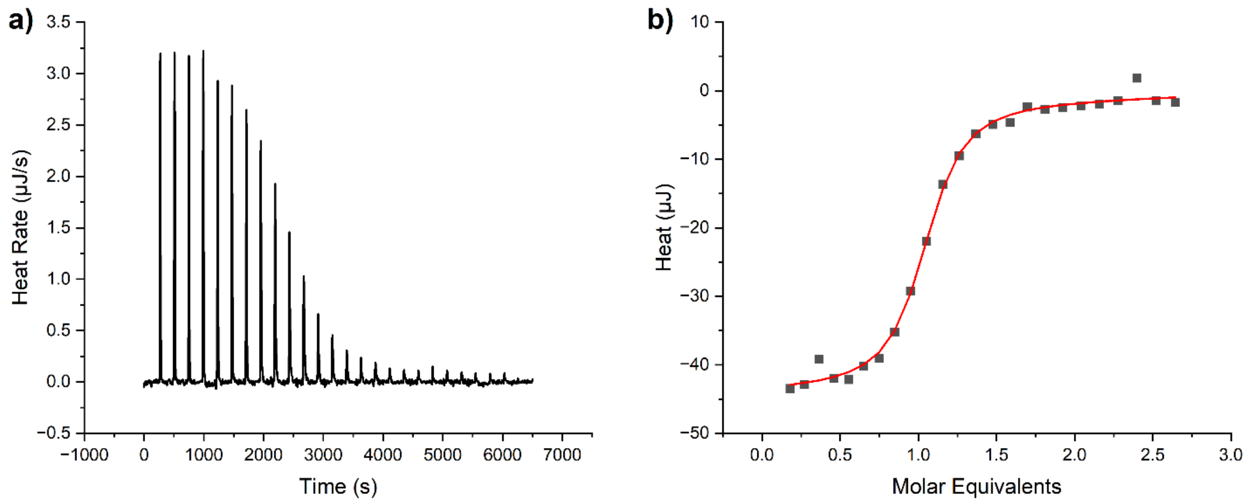

Figure S17. Isothermal titration calorimetry thermogram for addition of Gln to T72C-A126Y-GlnBP (a) and integrated heat per injection (•), fit using NanoAnalyze with an independent binding model plus a linear function to account for background heat released (—) (b).

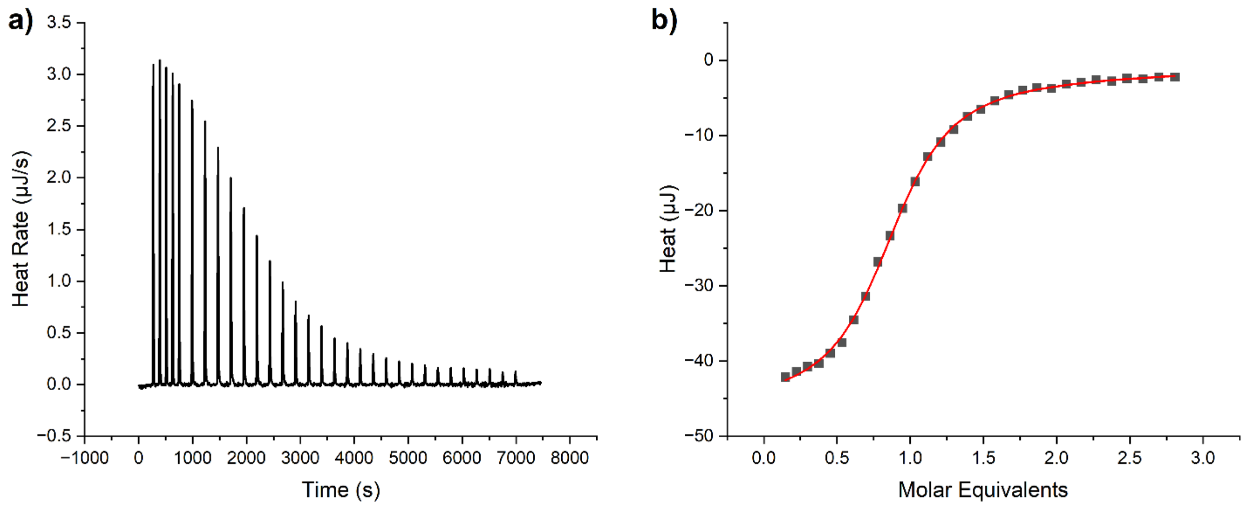

Figure S18. Isothermal titration calorimetry thermogram for addition of Gln to [Dy(DOTA-mal^{N})]$_{72}$-A$_{126}$Y-GlnBP (a) and integrated heat per injection (•), fit using NanoAnalyze with an independent binding model plus a linear function to account for background heat released (—) (b).

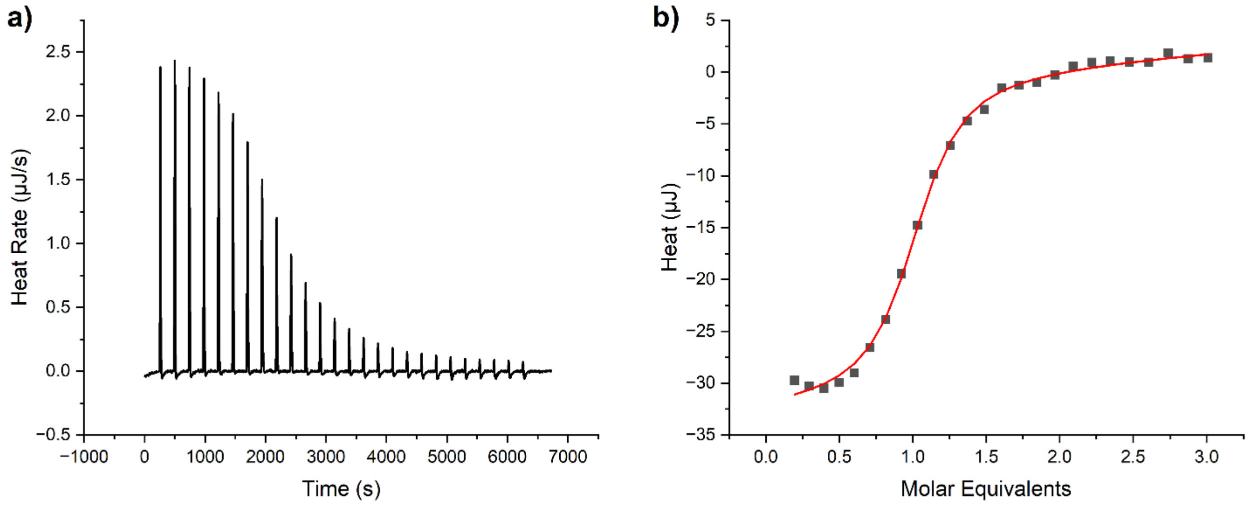

Figure S19. Isothermal titration calorimetry thermogram for addition of Gln to [Dy(DOTA-mal^{N})]$_{72}$-N$_{127}$Y-GlnBP (a) and integrated heat per injection (•), fit using NanoAnalyze with an independent binding model plus a linear function to account for background heat released (—) (b).

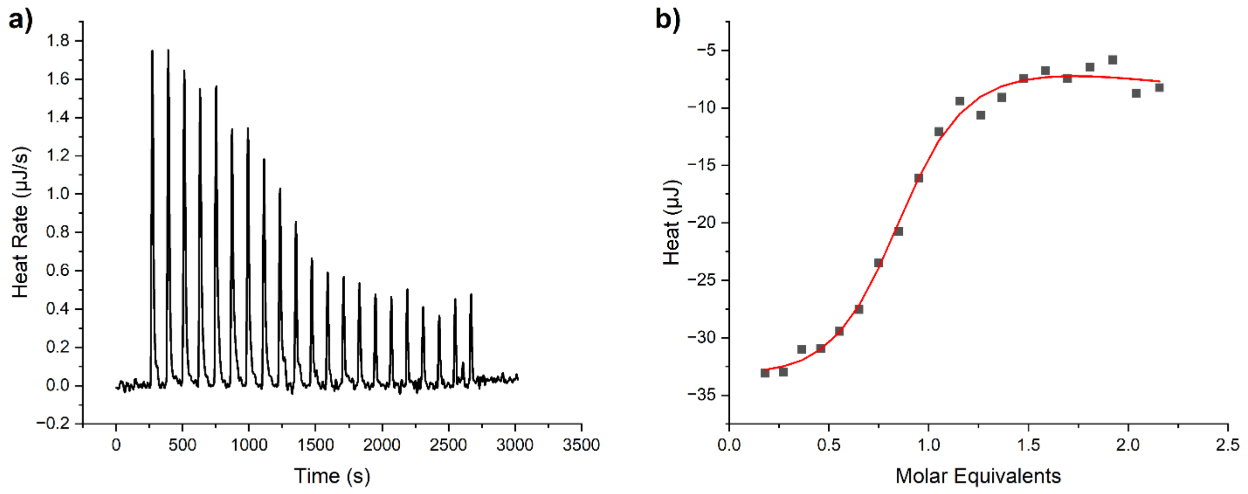

7Figure S20. Isothermal titration calorimetry thermogram for addition of Gln to $T_{72}C$-$Q_{183}Y$-GlnBP (a) and integrated heat per injection (•), fit using NanoAnalyze with an independent binding model plus a linear function to account for background heat released (—) (b).

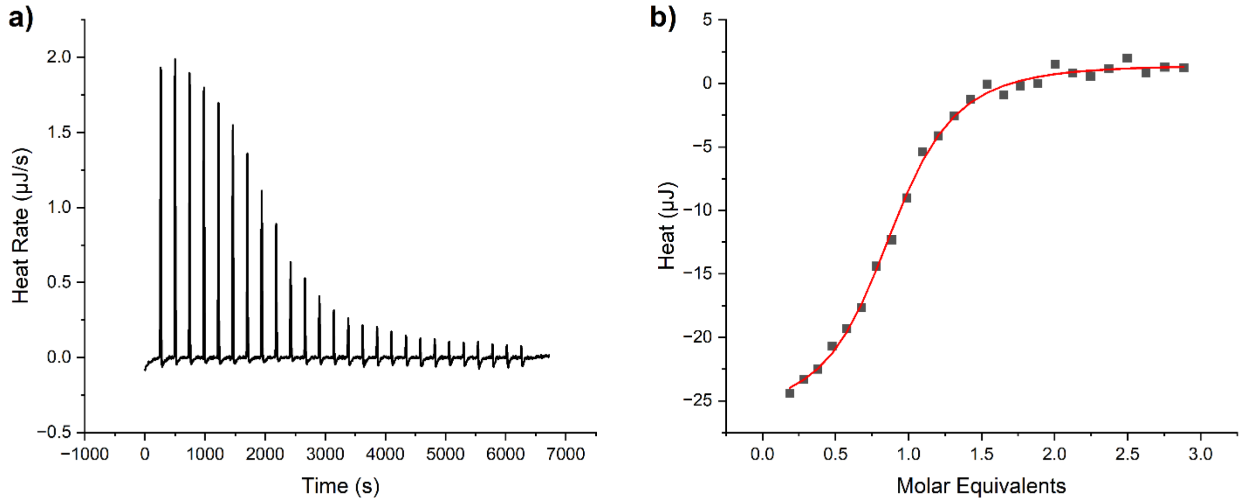

Figure S21. Isothermal titration calorimetry thermogram for addition of Gln to [Dy(DOTA-mal^{N})]$_{72}$-Q$_{183}$Y-GlnBP (a) and integrated heat per injection (•), fit using NanoAnalyze with an independent binding model plus a linear function to account for background heat released (—) (b).

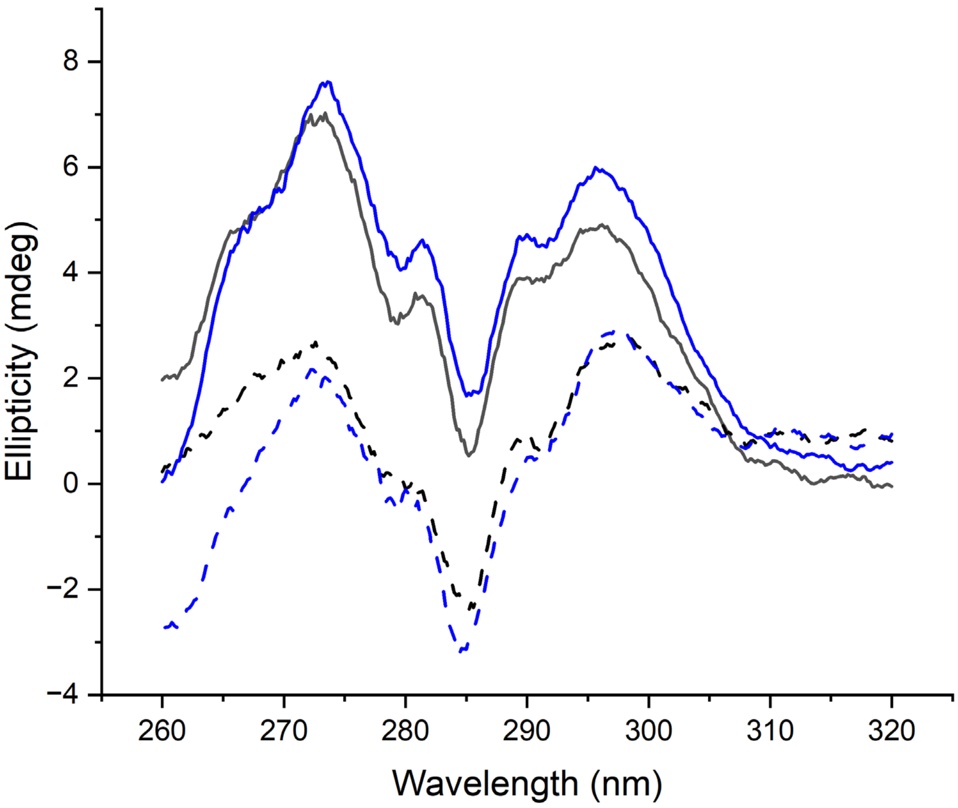

Figure S22. CD spectra for *apo*- (▬) and *holo*-$T_{72}C$-GlnBP (■ ■ ■), and apo- (▬) and *holo*-[Gd(DOTA-mal^{N})]$_{72}$-GlnBP (■ ■ ■) in PBS buffer, pH 7.4, 37 °C.

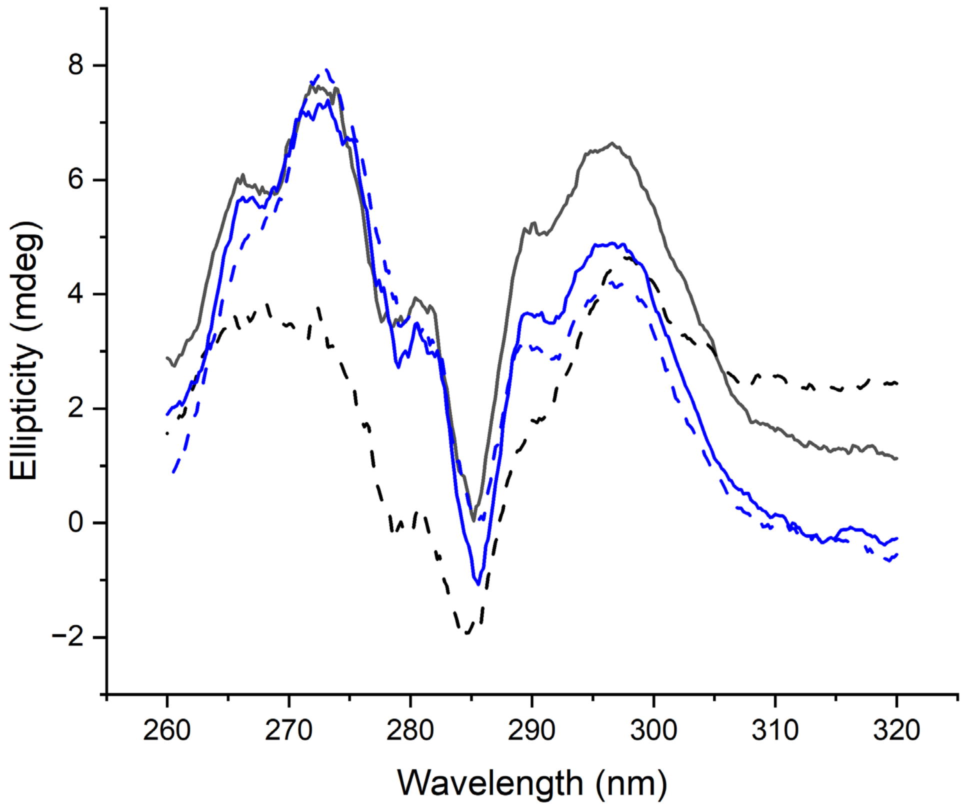

Figure S23. CD spectra of *apo*- (▬) and *holo*-N_{160}C-GlnBP (▪ ▪ ▪), and *apo*- (▬) and *holo*-[Gd(DOTA-mal^{N})]$_{160}$-GlnBP (▪ ▪ ▪) in PBS buffer, pH 7.4, 37 °C.

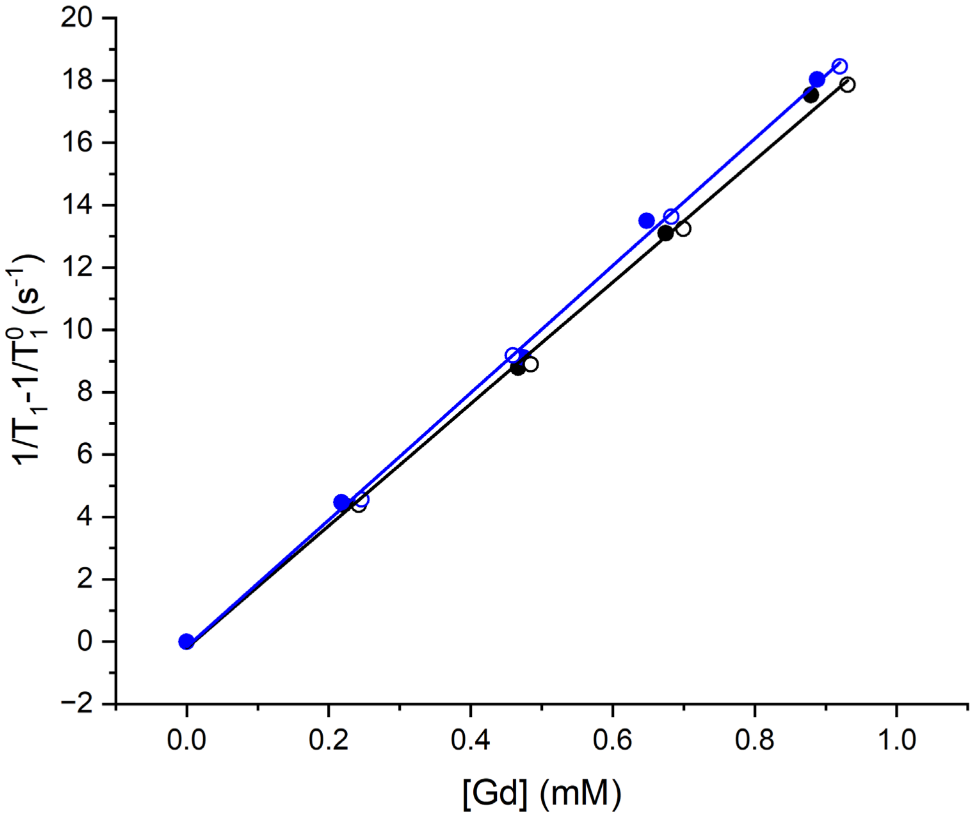

Figure S24. T_1 relaxivity data (•) and linear fit (—) for *apo*- (black) and *holo*- (blue) [Gd(DOTA-mal^N)]$_{72}$-GlnBP at 1.4 T. Open and filled circles represent replicate sets of experiments performed on independently prepared samples in PBS buffer, pH 7.4, 37 °C.

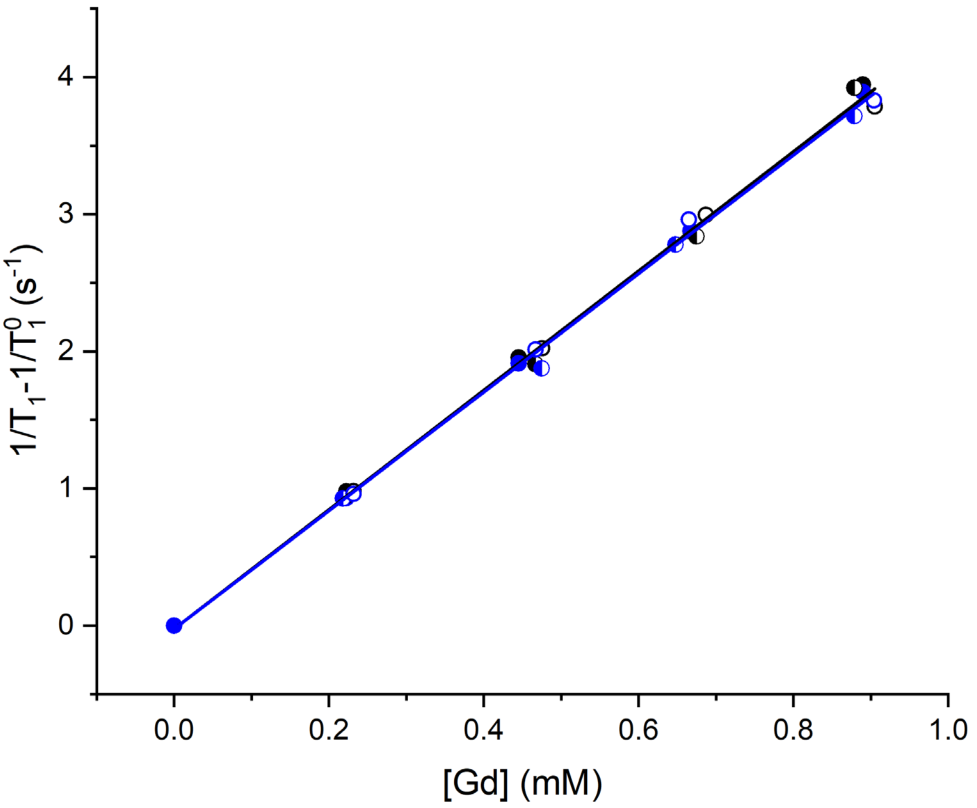

Figure S25. T_1 relaxivity data (•) and linear fit (—) for *apo*- (black) and *holo*- (blue) [Gd(DOTA-mal^N)]$_{72}$-GlnBP at 9.4 T. Open, filled, and half-filled circles represent replicate sets of experiments performed on independently prepared samples in PBS buffer, pH 7.4, 37 °C.

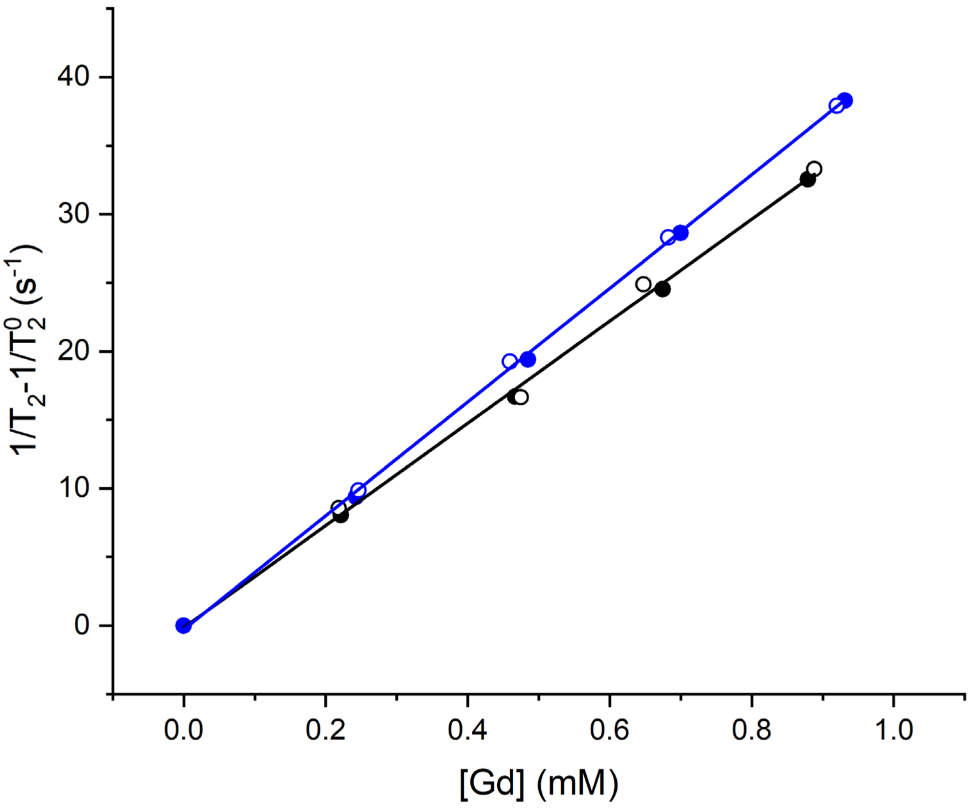

Figure S26. T_2 relaxivity data (•) and linear fit (—) for *apo*- (black) and *holo*- (blue) [Gd(DOTA-mal^N)]$_{72}$-GlnBP at 1.4 T. Open and filled circles represent replicate sets of experiments performed on independently prepared samples in PBS buffer, pH 7.4, 37 °C.

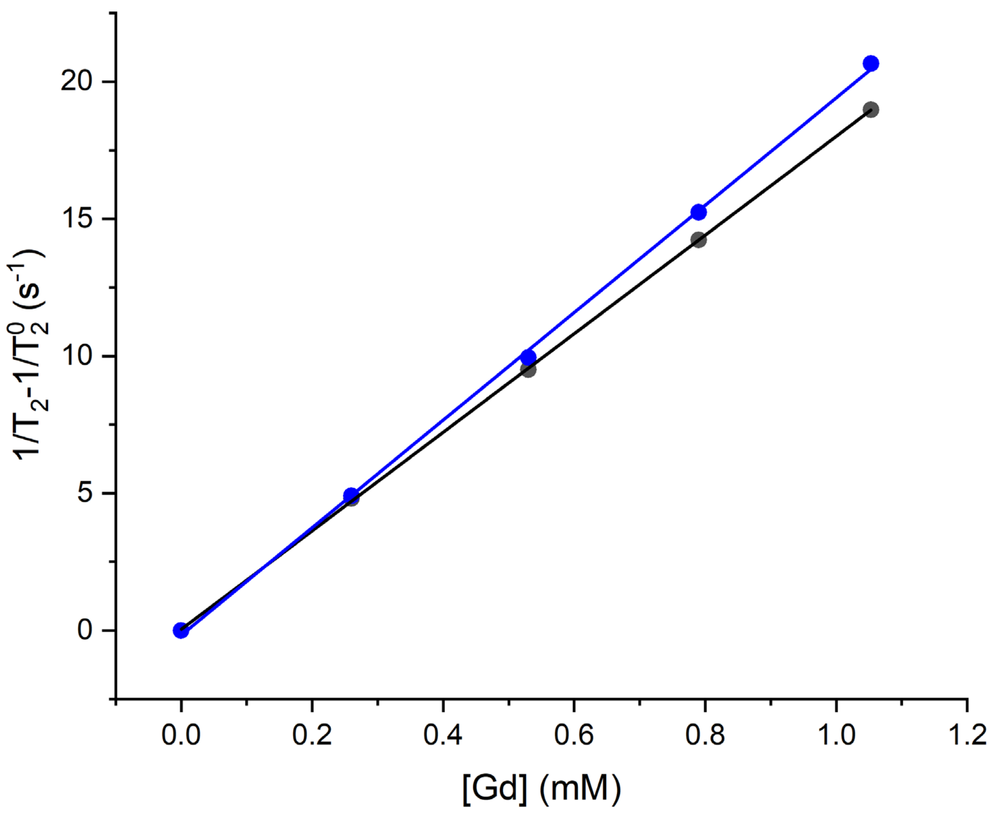

Figure S27. T_2 relaxivity data (•) and linear fit (—) for *apo*- (black) and *holo*- (blue) [Gd(DOTA-mal^N)]$_{122}$-GlnBP at 9.4 T. Experiment was performed in PBS buffer, pH 7.4, 37 °C.

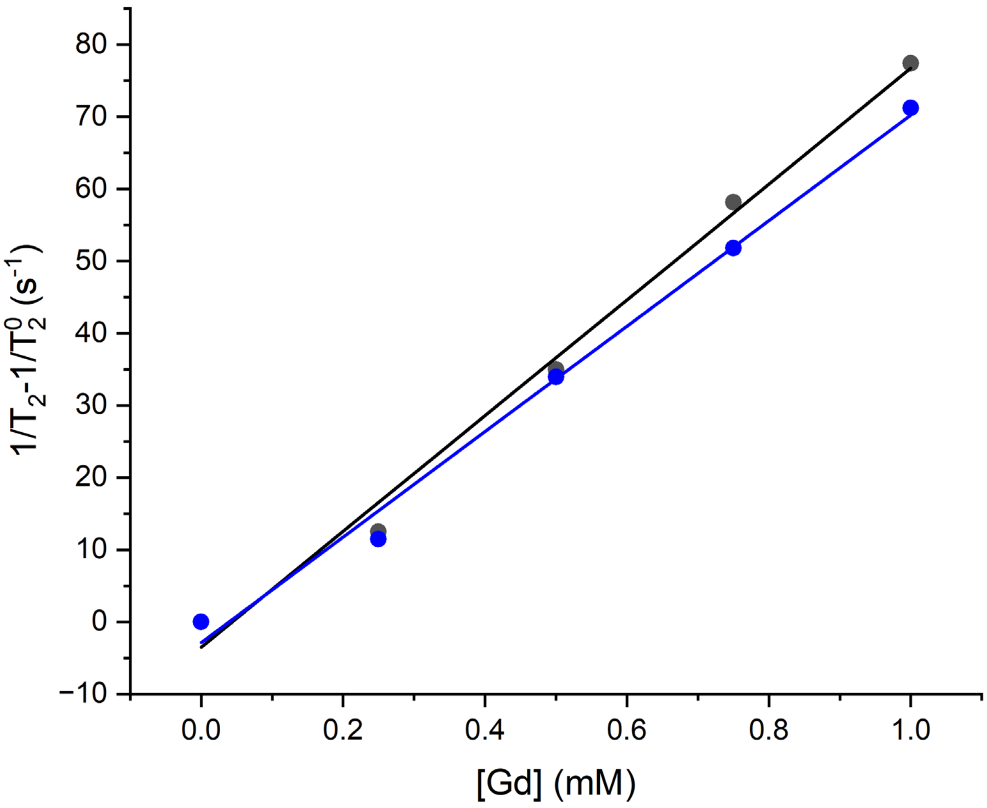

Figure S28. T_2 relaxivity data (•) and linear fit (—) for *apo*- (black) and *holo*- (blue) [Gd(DOTA-mal^N)]$_{160}$-GlnBP at 9.4 T. Experiment was performed in PBS buffer, pH 7.4, 37 °C.

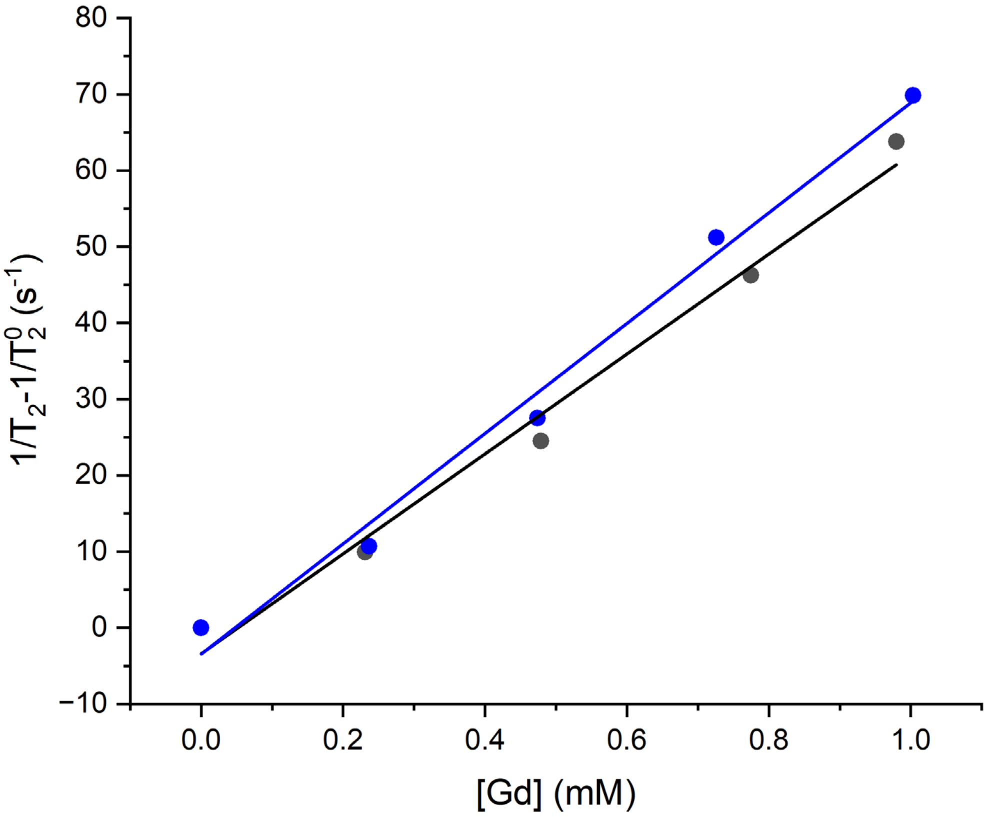

Figure S29. T_2 relaxivity data (•) and linear fit (—) for *apo*- (black) and *holo*- (blue) [Gd(DTPA-mal^N)]$_{72}$-GlnBP at 9.4 T. Experiment was performed in PBS buffer, pH 7.4, 37 °C.

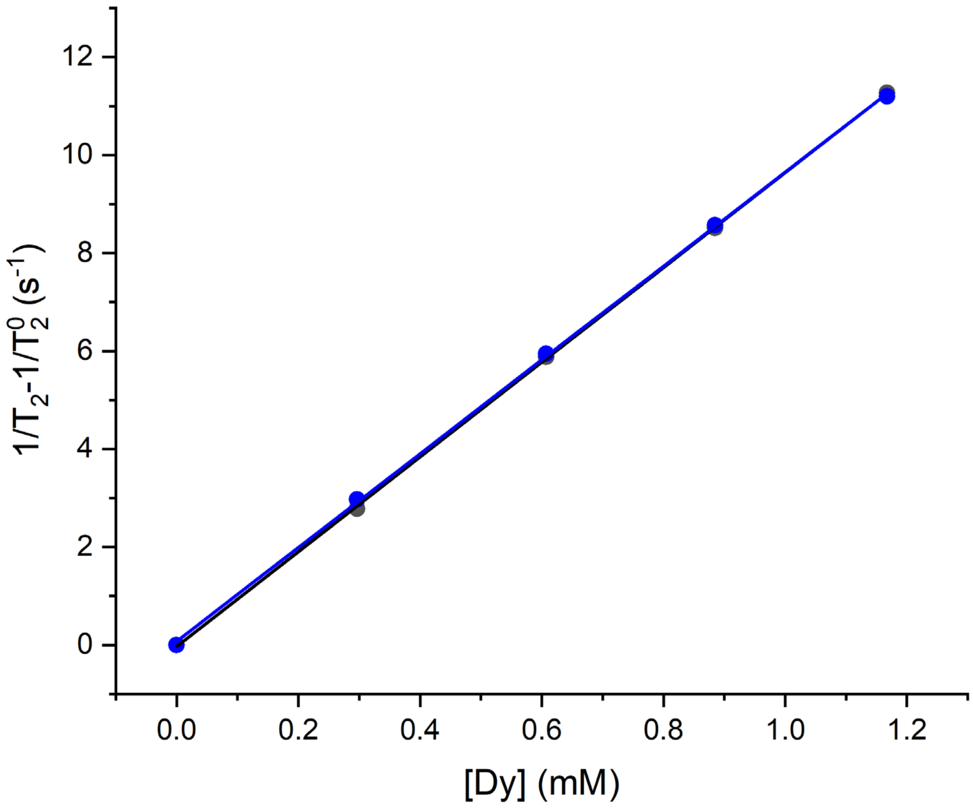

Figure S30. T_2 relaxivity data (•) and linear fit (—) for *apo*- (black) and *holo*- (blue) [Dy(DOTA-mal^N)]$_{72}$-A$_{126}$D-GlnBP at 9.4 T. Experiment was performed in PBS buffer, pH 7.4, 37 °C.

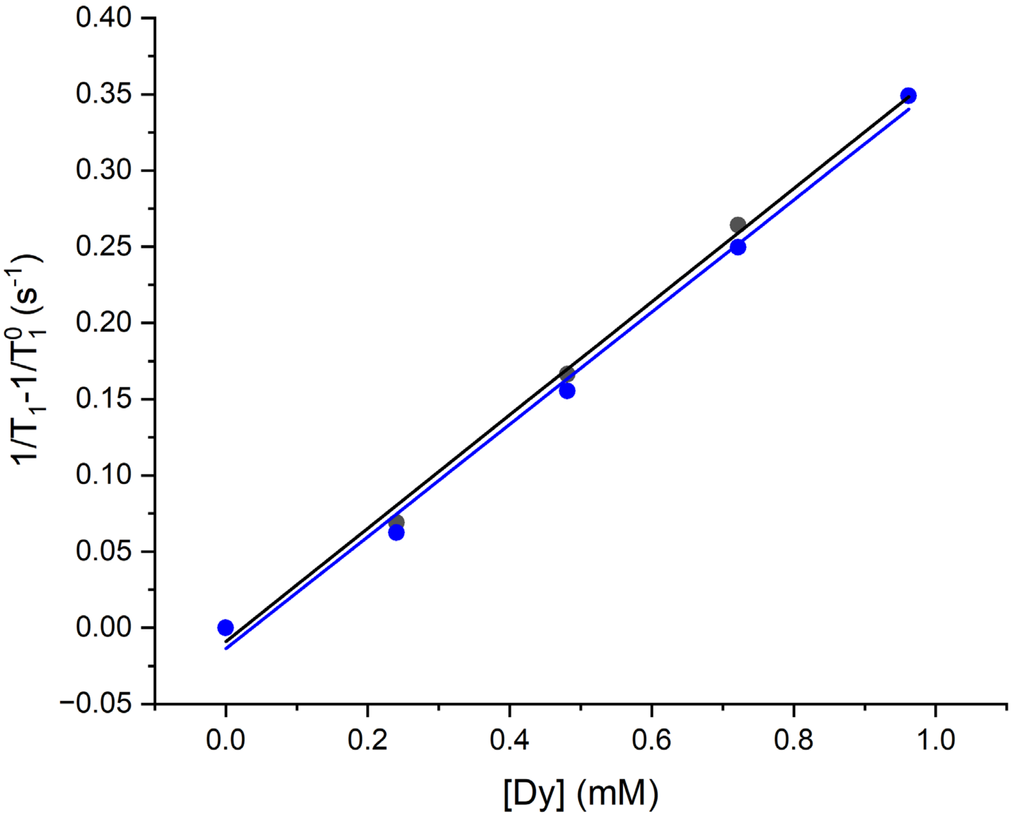

Figure S31. T_1 relaxivity data (•) and linear fit (—) for *apo*- (black) and *holo*- (blue) [Dy(DOTA-mal^N)]$_{72}$-GlnBP at 9.4 T. Experiment was performed in PBS buffer, pH 7.4, 37 °C.

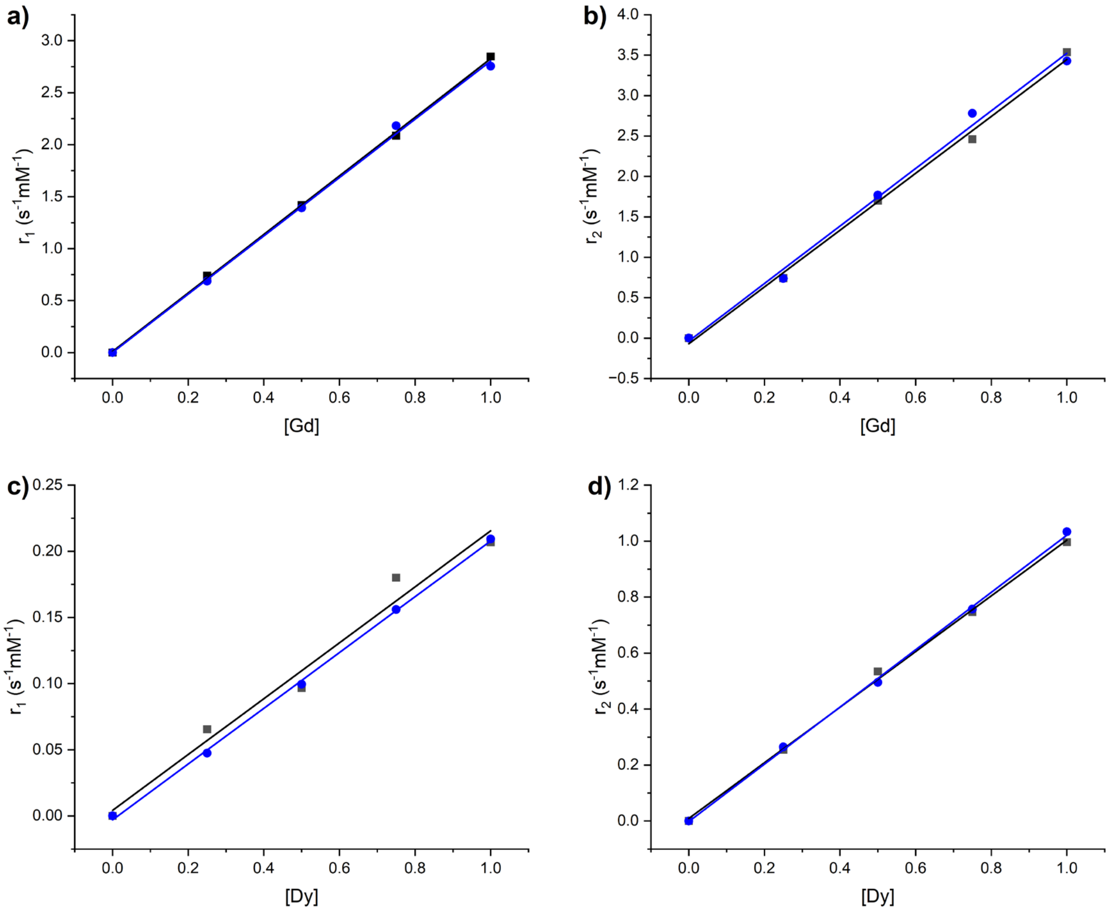

Figure S32. Relaxivity data (•) and linear fit (—) for [Ln(DOTA)(H_2O)] in the absence (black) or presence (blue) of Gln at 9.4 T. (a, b) Ln = Gd, T_1 and T_2, respectively; (c, d) Ln = Dy, T_1 and T_2, respectively. Experiments were performed in PBS buffer, pH 7.4, 37 °C.

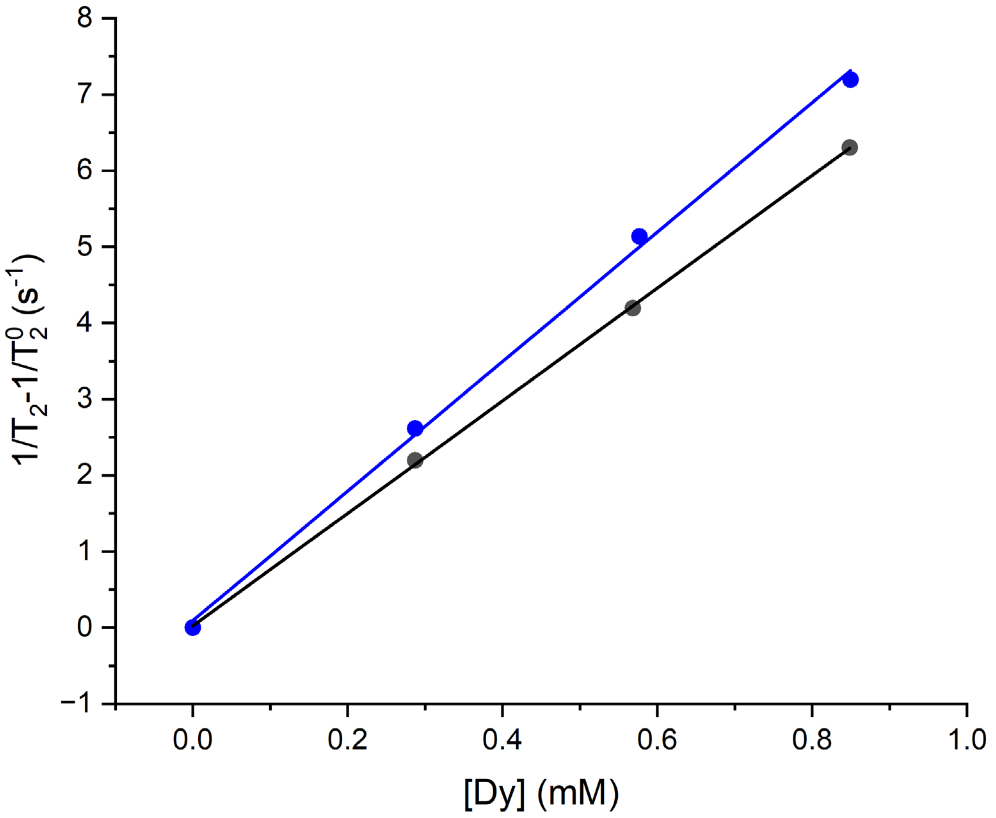

Figure S33. T_2 relaxivity data (•) and linear fit (—) for *apo*- (black) and *holo*- (blue) [Dy(DOTA-mal^{N})]$_{72}$-G$_{119}$K-GlnBP at 9.4 T. Experiment was performed in PBS buffer, pH 7.4, 37 °C.

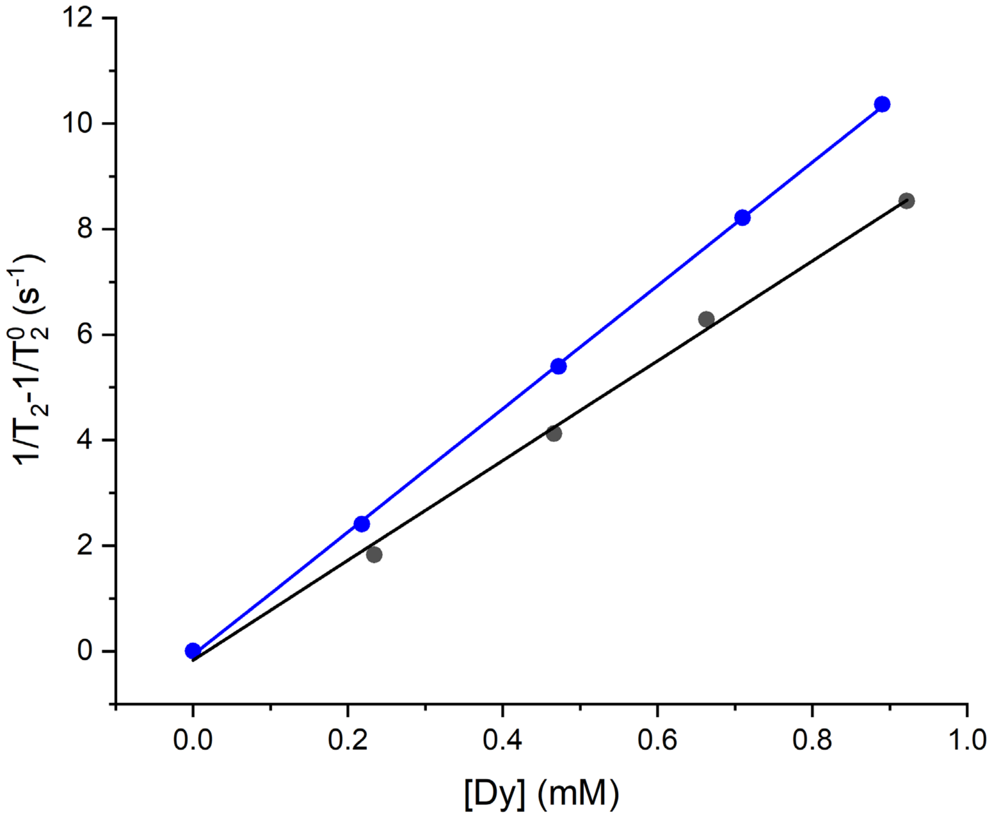

Figure S34. T_2 relaxivity data (•) and linear fit (—) for *apo*- (black) and *holo*- (blue) [Dy(DOTA-mal^N)]$_{72}$-A$_{126}$D-GlnBP at 9.4 T. Experiment was performed in PBS buffer, pH 7.4, 37 °C.

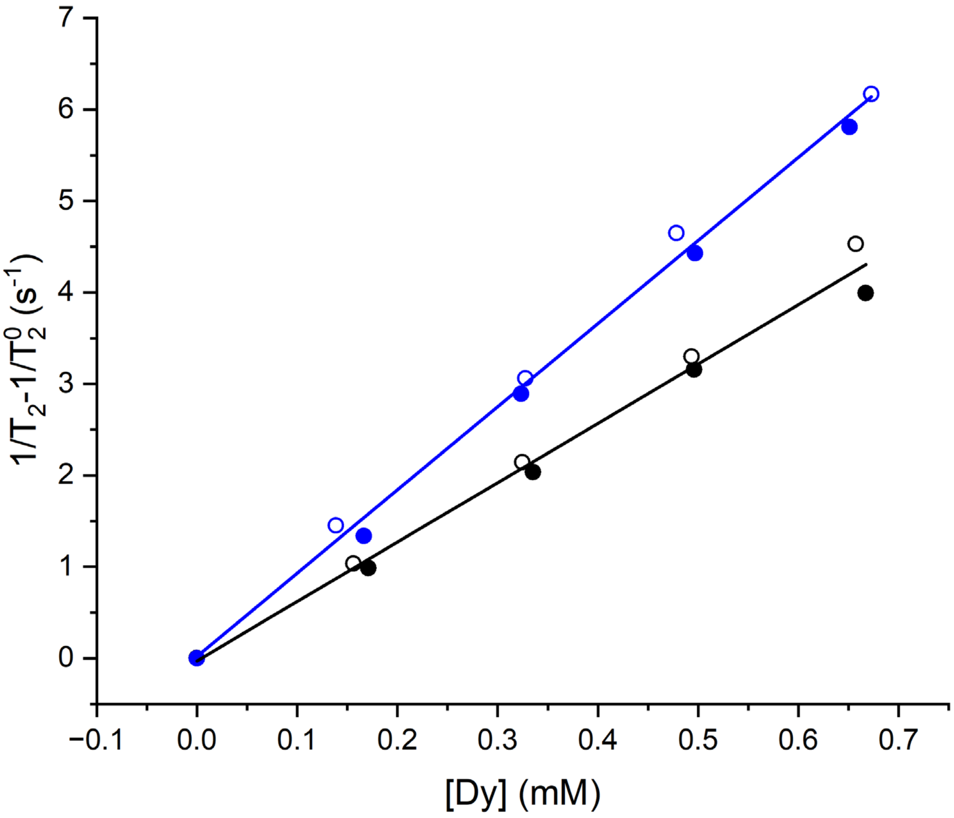

Figure S35. T_2 relaxivity data (•) and linear fit (—) for *apo*- (black) and *holo*- (blue) [Dy(DOTA-mal^N)]$_{72}$-N$_{127}$Y-GlnBP at 1.4 T. Open and filled circles represent replicate sets of experiments performed on independently prepared samples in PBS buffer, pH 7.4, 37 °C.

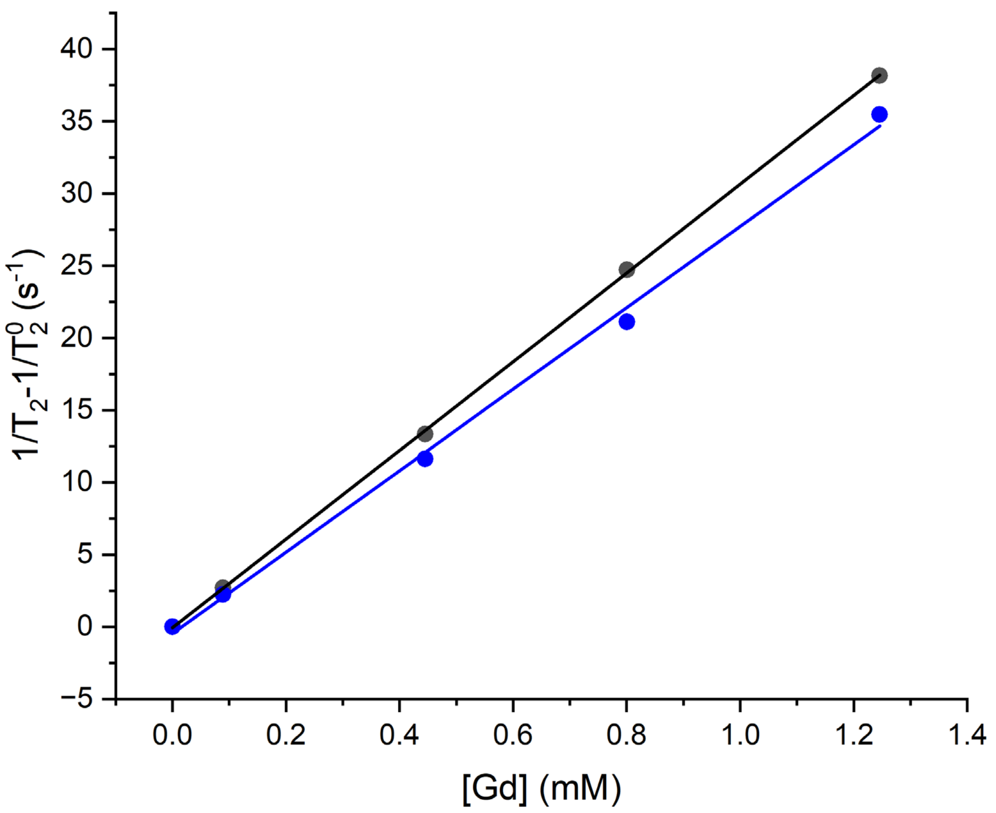

Figure S36. T_2 relaxivity data (•) and linear fit (—) for *apo*- (black) and *holo*- (blue) [Gd(DOTA-mal^{N})]$_{72}$-Q$_{183}$K-GlnBP at 9.4 T. Experiment was performed in PBS buffer, pH 7.4, 37 °C.

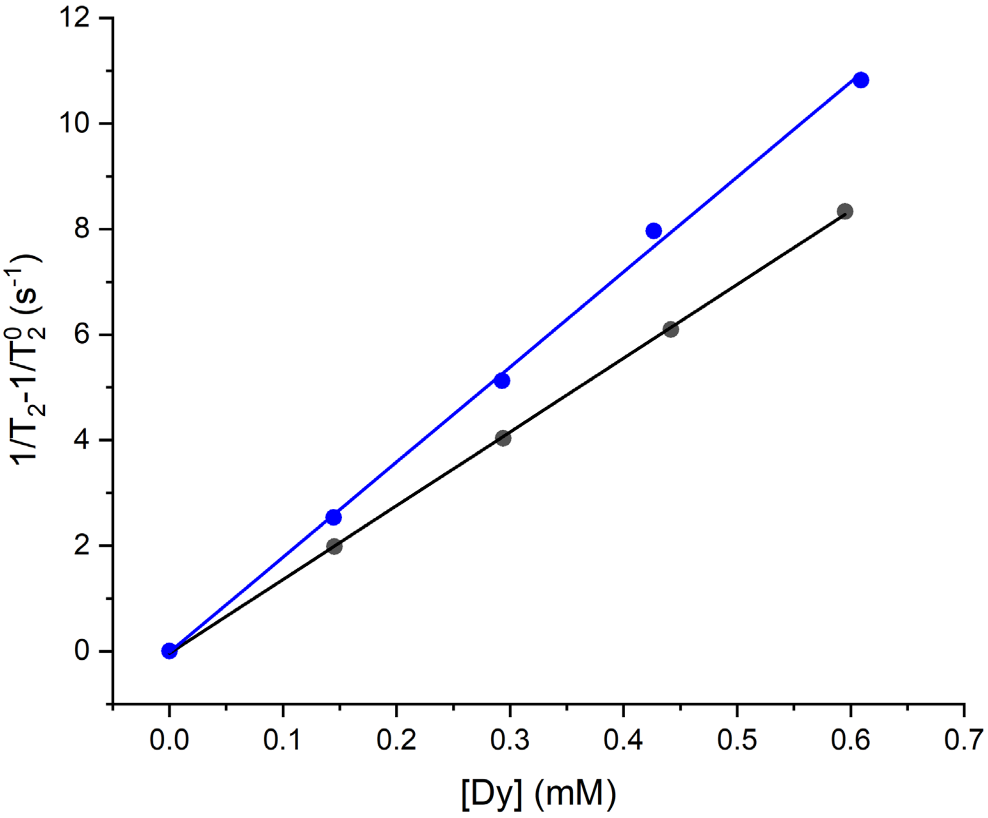

Figure S37. T_2 relaxivity data (•) and linear fit (—) for *apo*- (black) and *holo*- (blue) [Dy(DOTA-mal^N)]$_{72}$-Q$_{183}$Y-GlnBP at 9.4 T. Experiment was performed in PBS buffer, pH 7.4, 37 °C.

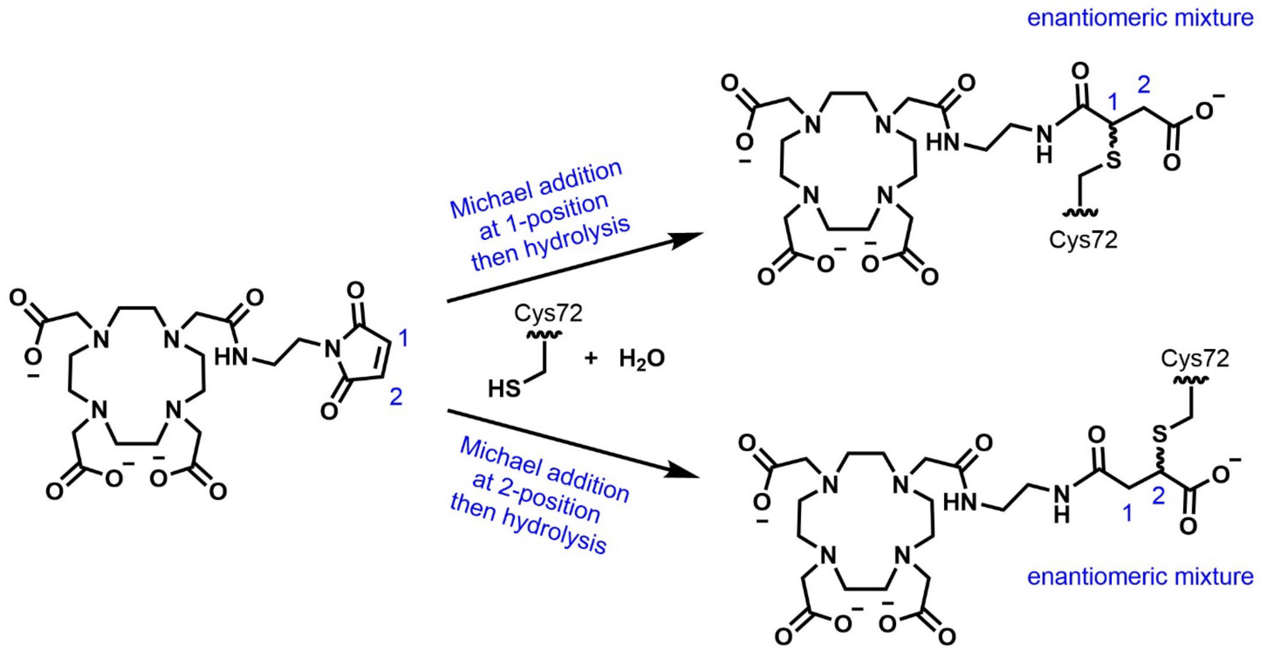

Figure S38. Bioconjugation of DOTA-mal^{N} necessarily produces a mixture of four isomeric products. These arise from Cys-ligation at either the 1- or 2- position of maleimide as marked above, and on either the 'top' or 'bottom face' of the olefin to produce two regioisomers, each in an unknown mixture of enantiomeric form.

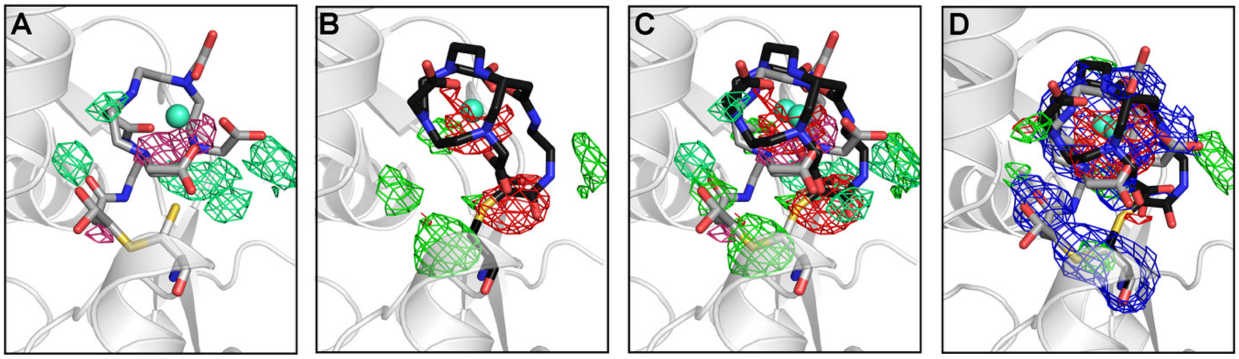

Figure S39. Alternative conformations of [Gd(DOTA-mal^{N})] in the crystal structure. (*A)* Conformation "A" of [Gd(DOTA-mal^{N})] (colored grey) in chain C refined as 100-percent occupancy with no alternative conformations. The ligand is positioned toward the left, with a positive difference map density shown on the right. (*B*) Conformation "B" of [Gd(DOTA-mal^{N})] (colored black) in chain C refined as 100-percent occupancy with no alternative conformations. The ligand is positioned toward the right, with positive difference map density shown on the left. (*C*) An overlay of both panels to show that the modeled left and right orientations occupy the observed positive difference density in panels A and B. In all panels the green and red maps correspond to F_O-F_C difference density maps contoured to $\pm$3.5 σ. (*D*) Conformation "A" and "B" of [Gd(DOTA-mal^{N})] in chain C are both modeled at 50-percent occupancy. 2F_O-F_C (blue) and F_O-F_C (green and red) electron density maps are contoured to 1 and $\pm$3.5 σ, respectively.

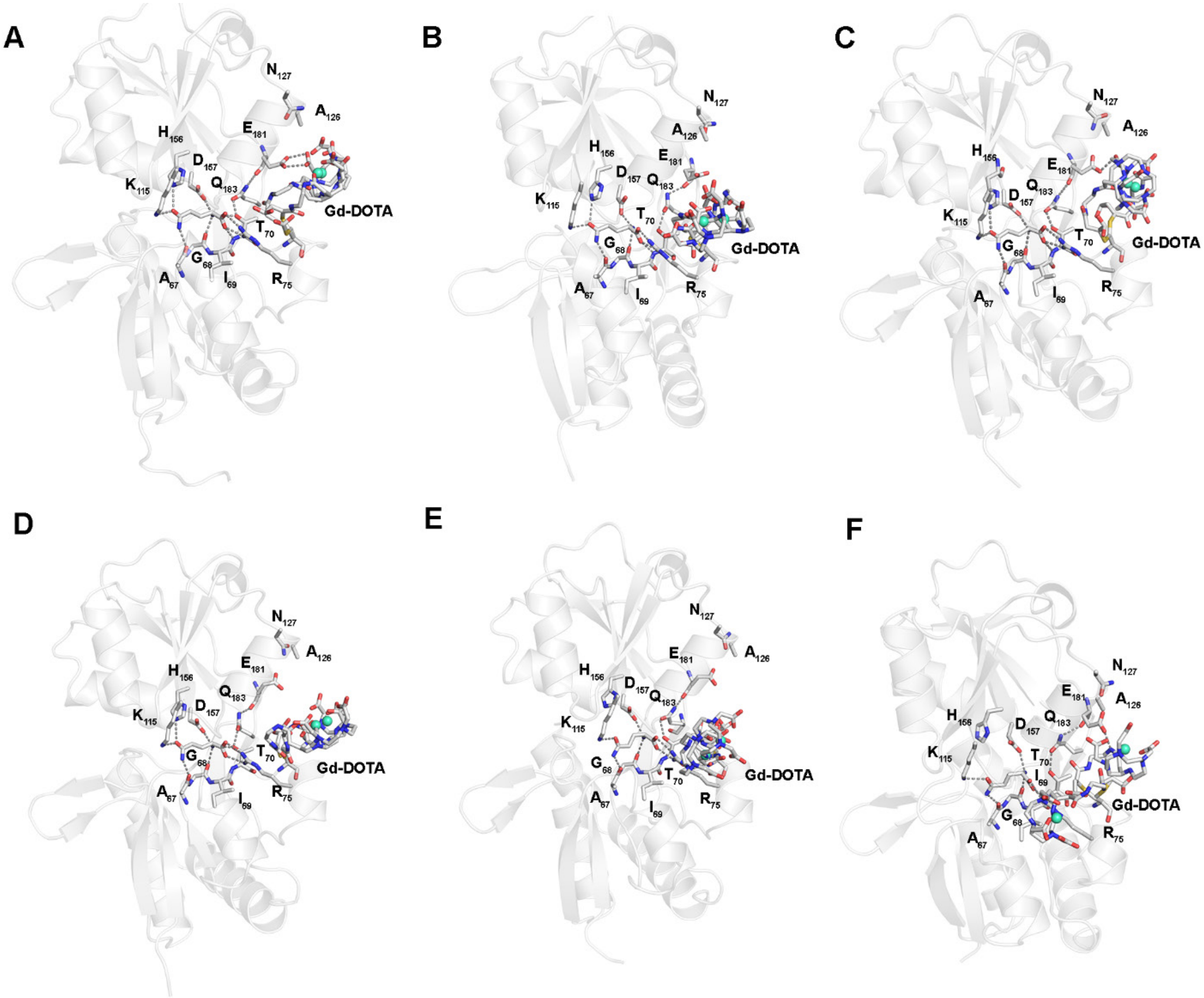

Figure S40: Hydrogen bonding network between bound glutamine and [Gd(DOTA-mal^{N})]. (*A-F)* Each respective chain found in the asymmetric unit of the crystal structure. In chains *A, C,* and *F* the hydrogen bonding network involved in the binding of glutamine extends to the [Gd(DOTA-mal^{N})] ligand. In all chains, the [Gd(DOTA-mal^{N})] is proximal to mutation-target residues A$_{126}$ and N$_{127}$. Five of the modeled metallocofactors are closer to A126 (6.8–7.9 Å, panels A, C, F) and seven are more distant (10.3–14.1 Å, panels B, D–F).

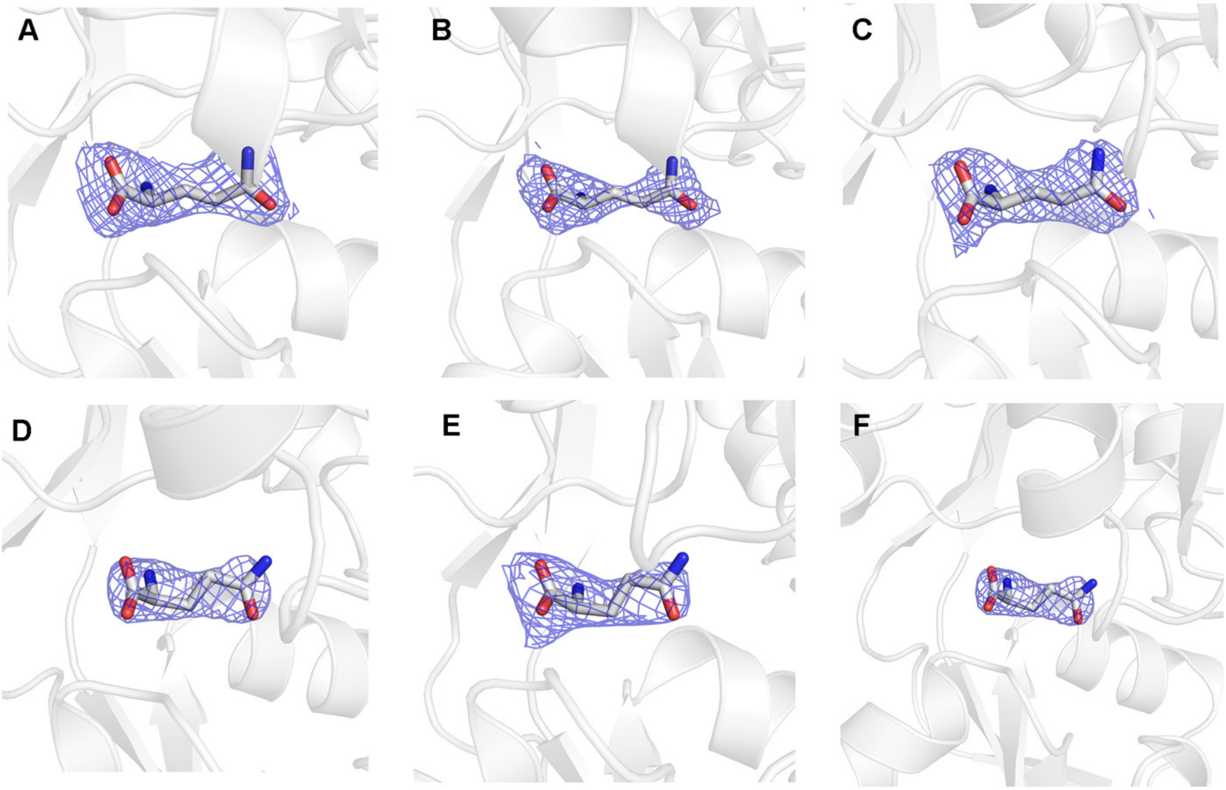

Figure S41. $2F_o$-F_c simulated annealing composite omit electron density maps of Gln bound in each chain of the holo structure. (A-F) Gln-bound in each respective chain found in the asymmetric unit of the crystal structure. All $2F_o$-F_c maps are contoured to 1σ.

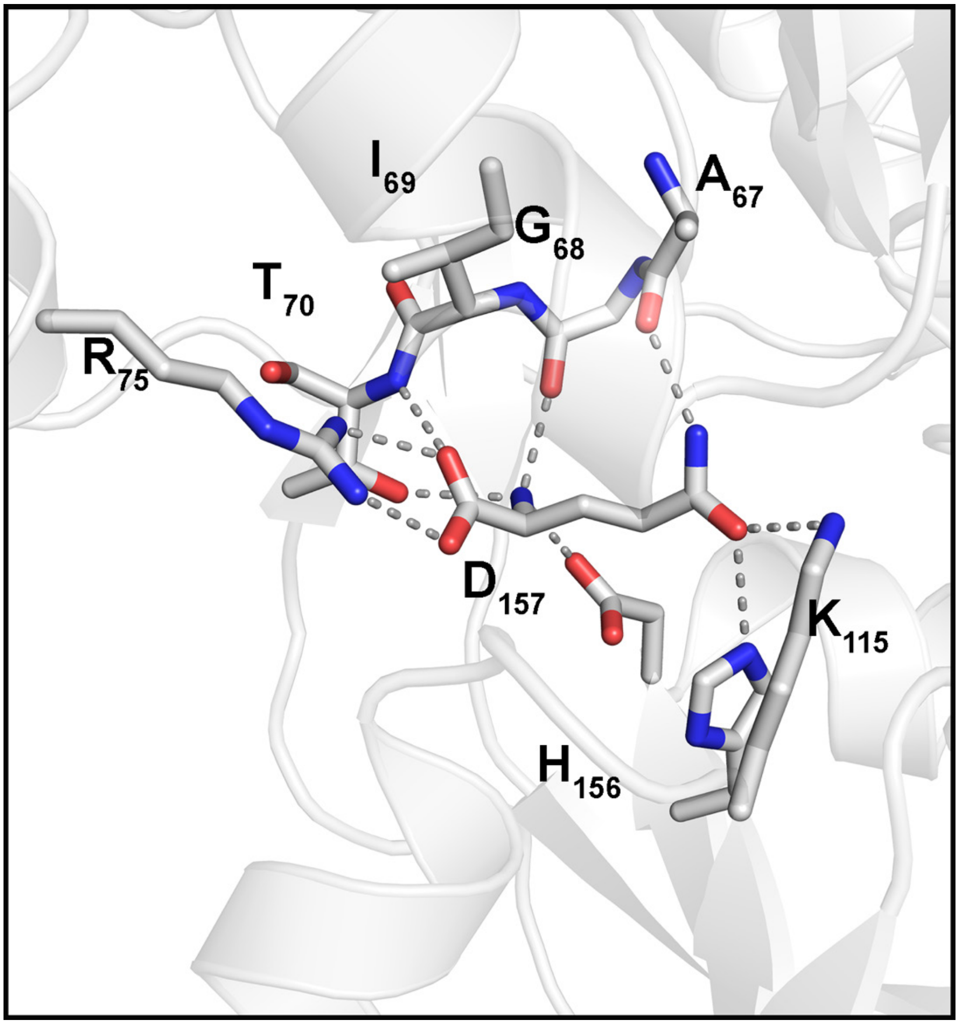

Figure S42. Hydrogen-bonding network of bound Gln in the *holo*-structure.

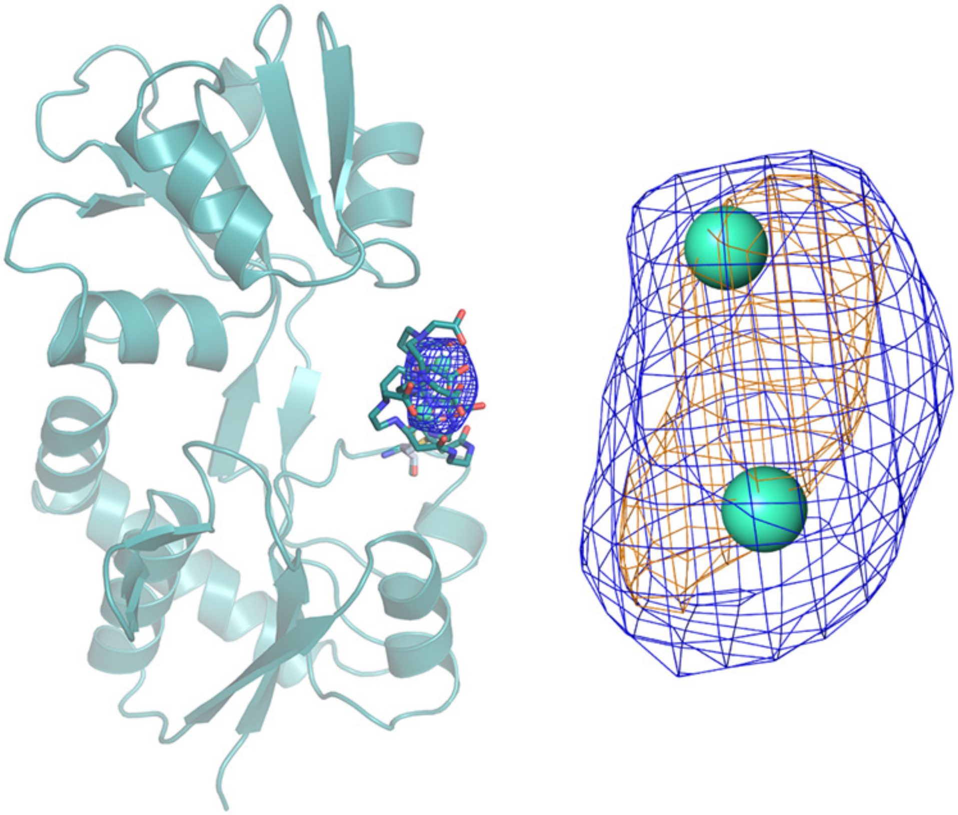

Figure S43. Overview of *apo*-structure with Gd^{3+} ion electron density in two different conformations (each conformation is modeled at 50-percent occupancy). Blue map: $2F_o$-F_c map contoured to 1σ. Orange map: Anomalous map contoured to 5σ.

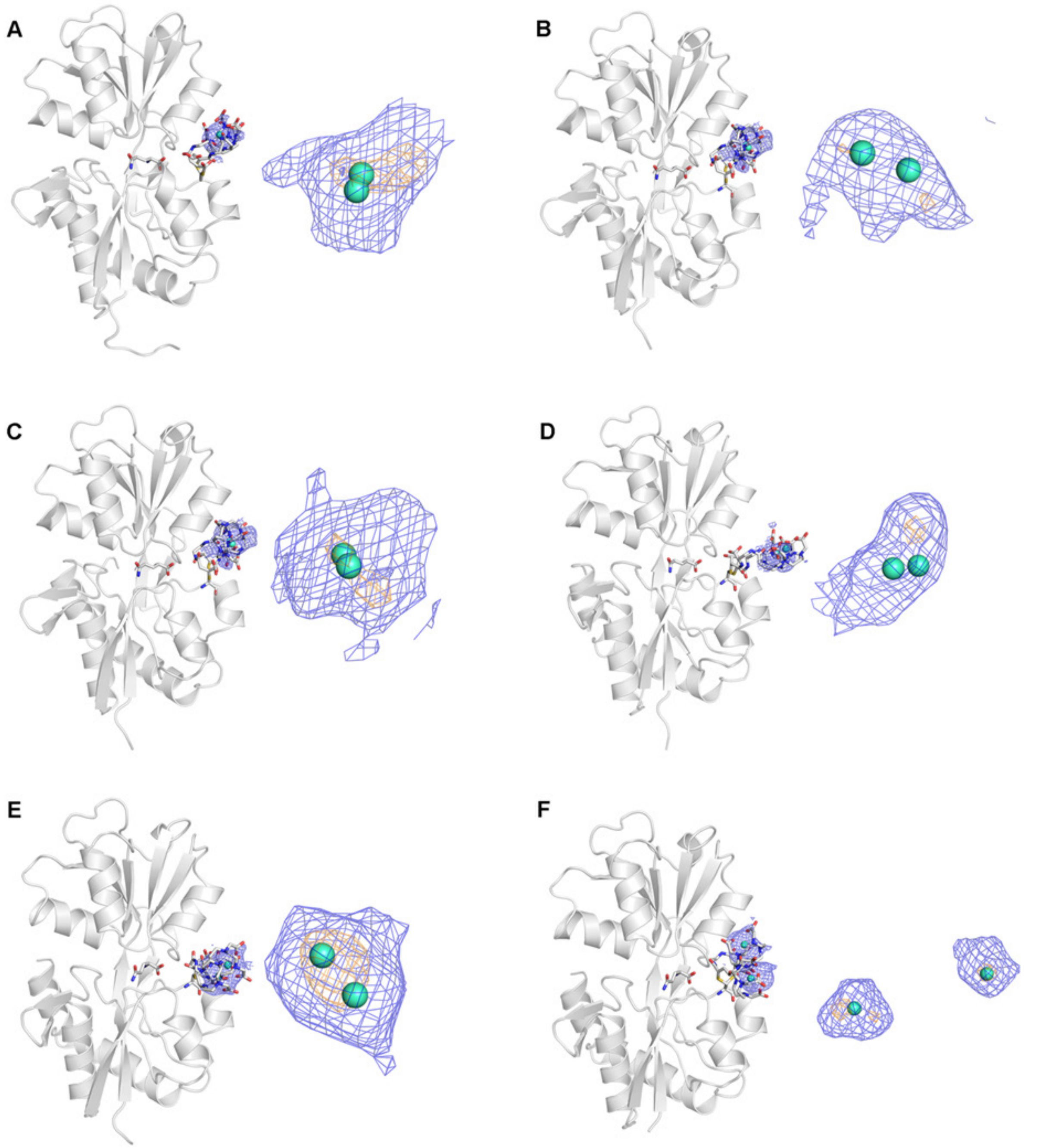

Figure S44. Overview of each chain in the *holo*-structure with Gd^{3+} ion electron density in two different conformations (each conformation is modeled at 50-percent occupancy). Blue map: $2F_o$-F_c map contoured to 1σ. Orange map: Anomalous map contoured to 5σ. (A-F) Each respective chain found in the asymmetric unit of the crystal structure.

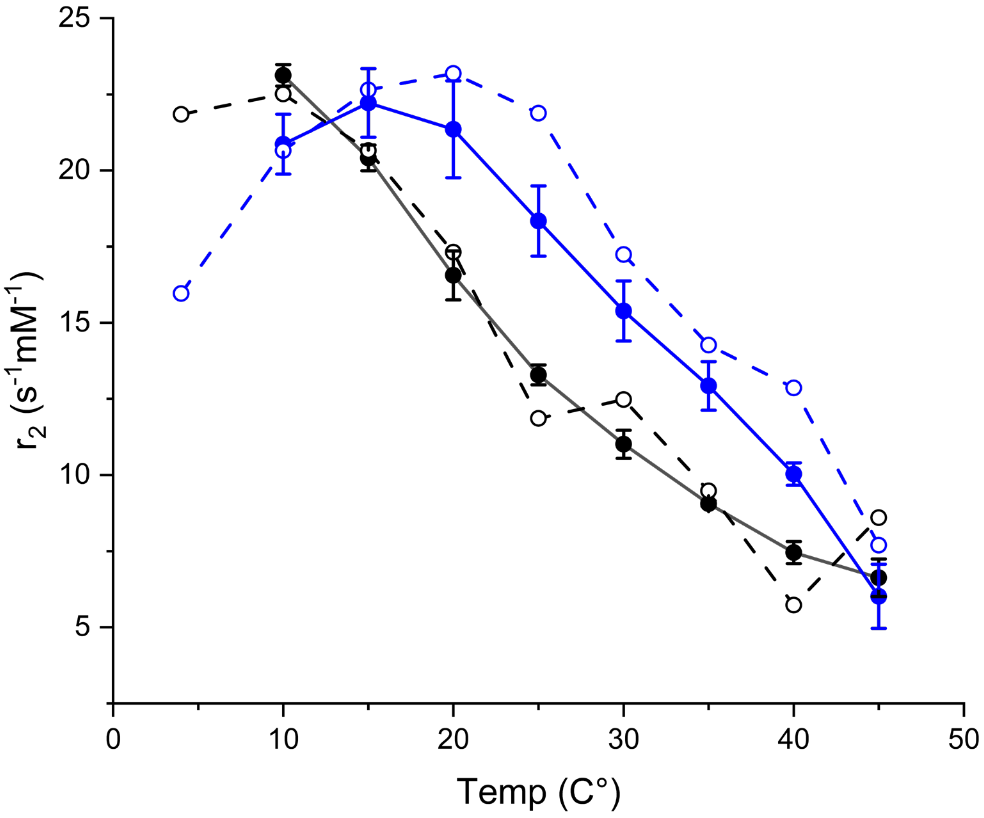

Figure S45. Variable-temperature relaxivity data for *apo*- (black) and *holo*- (blue) [Dy(DOTA-mal^{N})]$_{72}$-GlnBP (Filled circles and solid lines) and [Dy(DOTA-mal^{N})]$_{72}$-A$_{126}$Y-GlnBP (empty circles and dashed lines) at 11.8 T. Error bars represent one standard deviation for duplicate experiments. Experiments were performed in PBS buffer, pH 7.4.

References

(1) Tropea, J. E.; Cherry, S.; Waugh, D. S. Expression and Purification of Soluble His6-Tagged TEV Protease. In *High Throughput Protein Expression and Purification: Methods and Protocols*; Doyle, S. A., Ed.; Humana Press: Totowa, NJ, 2009; pp 297–307.

(2) Fatima, S.; Boggs, D. G.; Ali, N.; Thompson, P. J.; Thielges, M. C.; Bridwell-Rabb, J.; Olshansky, L. Engineering a Conformationally Switchable Artificial Metalloprotein. *J. Am. Chem. Soc.* **2022**, *144*, 21606–21616.

(3) Fatima, S.; Mehrafrooz, B.; Boggs, D. G.; Ali, N.; Singh, S.; Thielges, M. C.; Bridwell-Rabb, J.; Aksimentiev, A.; Olshansky, L. Conformation-Dependent Hydrogen-Bonding Interactions in a Switchable Artificial Metalloprotein. *Biochemistry* **2024**, *63*, 2040–2050.

(4) McPhillips, T. M.; McPhillips, S. E.; Chiu, H.-J.; Cohen, A. E.; Deacon, A. M.; Ellis, P. J.; Garman, E.; Gonzalez, A.; Sauter, N. K.; Phizackerley, R. P.; Soltis, S. M.; Kuhn, P. Blu-Ice and the Distributed Control System: Software for Data Acquisition and Instrument Control at Macromolecular Crystallography Beamlines. *J. Synchrotron Radiat.* **2002**, *9*, 401–406.

(5) Soltis, S. M.; Cohen, A. E.; Deacon, A.; Eriksson, T.; González, A.; McPhillips, S.; Chui, H.; Dunten, P.; Hollenbeck, M.; Mathews, I.; Miller, M.; Moorhead, P.; Phizackerley, R. P.; Smith, C.; Song, J.; van dem Bedem, H.; Ellis, P.; Kuhn, P.; McPhillips, T.; Sauter, N.; Sharp, K.; Tsyba, I.; Wolf, G. New Paradigm for Macromolecular Crystallography Experiments at SSRL: Automated Crystal Screening and Remote Data Collection. *Acta Crystallogr. D Biol. Crystallogr.* **2008**, *64*, 1210–1221.

(6) Cohen, A. E.; Ellis, P. J.; Miller, M. D.; Deacon, A. M.; Phizackerley, R. P. An Automated System to Mount Cryo-Cooled Protein Crystals on a Synchrotron Beamline, Using Compact Sample Cassettes and a Small-Scale Robot. *J. Appl. Crystallogr.* **2002**, *35*, 720–726.

(7) Russi, S.; Song, J.; McPhillips, S. E.; Cohen, A. E. The Stanford Automated Mounter: Pushing the Limits of Sample Exchange at the SSRL Macromolecular Crystallography Beamlines. *J. Appl. Crystallogr.* **2016**, *49*, 622–626.

(8) Kabsch, W. *XDS*. *Acta Crystallogr. D Biol. Crystallogr.* **2010**, *66*, 125–132.

(9) Brehm, W.; Triviño, J.; Krahn, J. M.; Usón, I.; Diederichs, K. *XDSGUI* : A Graphical User Interface for *XDS* , *SHELX* and *ARCIMBOLDO*. *J. Appl. Crystallogr.* **2023**, *56*, 1585–1594.

(10) Liebschner, D.; Afonine, P. V.; Baker, M. L.; Bunkóczi, G.; Chen, V. B.; Croll, T. I.; Hintze, B.; Hung, L.-W.; Jain, S.; McCoy, A. J.; Moriarty, N. W.; Oeffner, R. D.; Poon, B. K.; Prisant, M. G.; Read, R. J.; Richardson, J. S.; Richardson, D. C.; Sammito, M. D.; Sobolev, O. V.; Stockwell, D. H.; Terwilliger, T. C.; Urzhumtsev, A. G.; Videau, L. L.; Williams, C. J.; Adams, P. D. Macromolecular Structure Determination Using X-Rays, Neutrons and Electrons: Recent Developments in Phenix. *Acta Crystallogr. Sect. Struct. Biol.* **2019**, *75*, 861–877.

(11) McCoy, A. J.; Grosse-Kunstleve, R. W.; Adams, P. D.; Winn, M. D.; Storoni, L. C.; Read, R. J. Phaser Crystallographic Software. *J. Appl. Crystallogr.* **2007**, *40*, 658–674.

(12) Hsiao, C.-D.; Sun, Y.-J.; Rose, J.; Wang, B.-C. The Crystal Structure of Glutamine-Binding Protein fromEscherichia Coli. *J. Mol. Biol.* **1996**, *262*, 225–242.

(13) Emsley, P.; Lohkamp, B.; Scott, W. G.; Cowtan, K. Features and Development of Coot. *Acta Crystallogr. D Biol. Crystallogr.* **2010**, *66*, 486–501.

(14) Afonine, P. V.; Grosse-Kunstleve, R. W.; Echols, N.; Headd, J. J.; Moriarty, N. W.; Mustyakimov, M.; Terwilliger, T. C.; Urzhumtsev, A.; Zwart, P. H.; Adams, P. D. Towards Automated Crystallographic Structure Refinement with Phenix.Refine. *Acta Crystallogr. D Biol. Crystallogr.* **2012**, *68*, 352–367.

(15) Moriarty, N. W.; Grosse-Kunstleve, R. W.; Adams, P. D. Electronic Ligand Builder and Optimization Workbench (eLBOW): A Tool for Ligand Coordinate and Restraint Generation. *Acta Crystallogr. D Biol. Crystallogr.* **2009**, *65*, 1074–1080.

(16) Thompson, P. J.; Boggs, D. G.; Wilson, C. A.; Bruchs, A. T.; Velidandla, U.; Bridwell-Rabb, J.; Olshansky, L. Structure-Driven Development of a Biomimetic Rare Earth Artificial Metalloprotein. *Proc. Natl. Acad. Sci.* **2024**, *121* (33), e2405836121.

(17) *MolProbity: More and better reference data for improved all-atom structure validation - Williams - 2018 - Protein Science - Wiley Online Library*.

(18) PyMol Molecular Graphics System, Version 3.0 Schrödinger, LLC.

(19) Morin, A.; Eisenbraun, B.; Key, J.; Sanschagrin, P. C.; Timony, M. A.; Ottaviano, M.; Sliz, P. Collaboration Gets the Most out of Software. *eLife* **2013**, *2*, e01456.

(20) Raiford, D. S.; Fisk, C. L.; Becker, E. D. Calibration of Methanol and Ethylene Glycol Nuclear Magnetic Resonance Thermometers. *Anal. Chem.* **1979**, *51*, 2050–2051.

(21) Hahn, E. L. An Accurate Nuclear Magnetic Resonance Method for Measuring Spin-Lattice Relaxation Times. *Phys. Rev.* **1949**, *76*, 145–146.

(22) Meiboom, S.; Gill, D. Modified Spin-Echo Method for Measuring Nuclear Relaxation Times. *Rev. Sci. Instrum.* **1958**, *29*, 688–691.
